# A Closed-Loop Measurement Study of Runtime Governance in AI-Driven Smart Building Climate Control

**DOI:** 10.3390/s26154921

**Published:** 2026-08-04

**Authors:** Norkobil Saydirasulov Saydirasulovich, Dilmurod Abdujalilovich Davronbekov, Makhmudov Makhsum Mubashirovich, Young Im Cho

**Affiliations:** 1 Department of Computer Engineering, Gachon University, Sujeong-gu, Seongnam-si 461-701, Gyeonggi-do, Republic of Korea; saydirasulov@gachon.ac.kr; 2 Department of Engineering and Communications, ZARMED University, Samarkand Campus, Khodja Akhror Vali Street 23, Samarkand 140100, Uzbekistan; 3 Department of Technologies of Mobile Communication, Tashkent University of Information Technologies Named After Muhammad Al-Khwarizmi, Tashkent 100084, Uzbekistan; d.davronbekov@gmail.com; 4 Scientific and Innovation Center of Information and Communication Technologies “UNICON.UZ” LLC, Tashkent 100187, Uzbekistan; maxmudovunicon@gmail.com

**Keywords:** AI governance, IoT runtime governance, closed-loop testbed, admission control, rollback, residual risk, safety oracle, distribution shift, robustness analysis, reproducibility

## Abstract

Which runtime governance mechanisms reduce physical risk when a learned controller drives a building’s climate, and under what conditions? We develop a closed-loop software-in-the-loop testbed in which a setpoint model trained on real occupancy data drives a physics-based thermal zone through a declarative governance plane, with outcomes scored by an independent safety oracle, and we run the same governance logic on a real MQTT stack with an in-process policy decision point and a hash-chained audit log. Under distribution shift, admission control reduces unsafe physical exposure by 19.4%, from 1185.3 to 954.8 °C·min, whereas adding checkpoint rollback reduces it by only a further 0.2% in the reference run (0.1–0.4% across sensor noise seeds): the governance decision takes 0.44 ms while physical recovery takes a median of 61 min. Prevention therefore outperforms recovery in the studied thermal system, and the remaining avoidable exposure is driven by the policy’s estimate of occupancy context. A deterministic single-rule thermostat incurs 50% more exposure under shift while the learned controller uses a 38% higher heating demand proxy: a safety–demand trade-off, not evidence that learned control is necessary. A plant sweep yields an operating envelope criterion for inertial plants: rollback contributes materially to safety only when the plant is restored before the next command arrives and sampled before it can leave the safe set; on slower plants it removes at most 14.7%, with a transition band in between. No physical hardware was operated.

## 1. Introduction

This paper studies one control loop concretely: a learned setpoint controller, trained on real occupancy data, whose commands must pass an executable governance plane before reaching a physics-based thermal zone. The mechanisms under study (policy-gated admission, checkpoint rollback and hash-linked audit) are not unique to building control, but **every quantitative claim in this paper is evaluated within this smart building climate control scenario**, and the one result transferred beyond it (the operating envelope criterion of Section 9) is obtained by an explicit parameter sweep over a stated class of inertial thermal models rather than assumed to hold universally. In sensor-driven IoT deployments, including smart buildings, environmental monitoring networks, and industrial process control, sensor observations trigger AI-driven actuation decisions that affect physical environments. Temperature sensors, occupancy detectors, air quality monitors, and smart meter readings generate continuous data streams that must pass through governance checks before triggering control actions. These difficulties arise not only from model accuracy limitations but also from fragmented orchestration, weak runtime observability, inconsistent rollback behavior, and the absence of executable governance mechanisms that can validate sensor metadata integrity before admitting actuation decisions.

Emerging governance frameworks such as the NIST AI Risk Management Framework and ISO/IEC 42001 emphasize accountability, traceability, and continuous oversight [1,2]. Yet these frameworks do not prescribe how governance rules should be executed inside IoT runtime systems. In many deployments, governance remains a checklist or compliance artifact rather than an operational component that can admit, throttle, reject, or roll back runtime actions. This gap is especially consequential in AI-driven IoT systems, where errors may affect physical processes and human environments.

This paper addresses the problem from a systems instrumentation perspective. The object of study is the governance runtime, not the AI: “AI-driven” refers to the deployment context in which inference services participate in the sensor-to-actuator loop, and the AI service functions as a realistic generator of control intents, some of which are wrong. The governance layer is intentionally deterministic, because in a safety- and audit-oriented setting an admit-or-reject decision must be reproducible and inspectable rather than learned. The central idea is to make governance executable through runtime policy gates, state machine semantics, checkpointed rollback, and auditable traces, then to measure what each mechanism is worth once its decisions have physical consequences.

The testbed implements four policy gates: safety and operational constraints (G1), privacy and data-handling constraints (G2), resilience and runtime health constraints (G3), and auditability constraints (G4). Incoming intents are admitted, throttled, rejected, or rolled back according to these gates. A governance state machine defines admission, execution, commit, throttling, rollback, and failed safe states. This design makes the governance behavior inspectable and reproducible.

This paper does not claim a new policy language, a new automatic verifier, a production-ready IoT platform, or deployment-level validation. Mature systems such as Soteria [3], IoTSan [4], IoTGuard [5], IoTSafe [6], iRuler [7], HomeGuard [8], and TAPInspector [9] address substantially deeper problems in IoT policy gap discovery, rule interaction analysis, safety verification, and runtime enforcement. Our contribution is narrower and empirical: we close the control loop through a physics-based plant model and measure which of these mechanisms actually reduces physical risk, and under what conditions. The governance overhead, queue pressure, and policy gap characterizations that a systems paper would foreground are reported as supporting material (Appendix B and Appendix C); the spine of this paper is the closed-loop finding.

The central finding of this paper is negative, and it inverts the ordering the governance literature commonly assumes. We built a closed loop in which governance decisions have physical consequences and measured what each mechanism buys. Admission control reduces unsafe physical exposure substantially; checkpoint rollback, added on top, removes almost none of it and does not reduce the peak excursion, because the room recovers far more slowly than the decision that governs it. What removes exposure is not recovering from the bad command but never admitting it, and how much a deployable policy can prevent is limited by its estimate of context rather than by the runtime.

Concretely, we provide:

1. **A closed-loop (SIL) testbed in which governance decisions have physical consequences.** A real setpoint model, trained on a real occupancy dataset, drives a physics-based thermal plant through the governance plane; because the plant has inertia, a rejected intent and an admitted-then-rolled-back intent differ measurably. Each arm is scored by an independent safety oracle that the admission gates never receive and cannot influence, which makes the false negative measurements non-circular; Section 3.6 states the one place in the recovery path where this testbed is idealized.
2. **An automated, oracle-based policy gap finder.** It mutates the declarative gates and generates coverage-guided control intents against an independent physical safety oracle (a metamorphic relation), automatically ranking the physical exposure each policy omission admits and flagging the shipped policy’s own gap; policy testing without a physical oracle detects none of these physical actuation gaps.
3. **A measurement, in physical units, of what each mechanism buys.** Under distribution shift, admission control cuts unsafe exposure from 1185.3 to 954.8 °C·min; checkpoint rollback removes a further 0.2% (0.1–0.4% across sensor noise seeds) and does not reduce the peak; one context predicate reaches 727.7 and an oracle predicate 1.2. The ordering is the finding, and it holds across six model architectures, four seeds, three sensor noise levels and four safety bands.
4. **A deployment criterion for runtime rollback.** A speed sweep over more than two orders of magnitude shows a threshold rather than a gradient: rollback removes about 95% of unsafe exposure on plants restorable faster than the command interval, and at most 14.7% on those that are not (4.8% at a fast, 1 s monitor). Both bounds are properties of the deployment, measurable before any code is written.
5. **Rejection semantics dominate rejection accuracy.** An oracle-accurate gate that reverts to a context-blind held state (924.8 °C·min) is outperformed by a less accurate gate that reverts to a context-independent safe state (727.7): what a gate does when it rejects can matter more than how often it is right.
6. **A reviewable artifact.** The governance plane runs on a real MQTT bus with an in-process executable policy decision point and a hash-chained audit log (decision latency 0.44 ms, measured not modeled); the same gates are also measured through a real OPA server over HTTP (0.94 ms). The testbed, trained model, all experiments, 25 tests, and a hardware abstraction layer with a complete Raspberry Pi backend are released and reproduced on three computational environments spanning Windows 10, Windows 11 and Ubuntu 22.04.

We state one limitation alongside the contributions, because it bears on how every number should be read: **we stop at Tier 3. No physical sensor or actuator hardware was operated**, so the closed loop of this paper is software-in-the-loop and not hardware-in-the-loop, and we use the terms in their standard cyber-physical sense (Section 5). Every physical quantity reported here is produced by a digital twin whose model and parameters are given in full in Section 8.1, so that they can be challenged rather than trusted. The released hardware abstraction layer is complete on both sides, and a mechanical parity check verifies that the governance code path is identical in simulation and on the Raspberry Pi. What remains is to run it, and that is future work rather than a claim.

We organize the study around three research questions.

**RQ1**  Can adversarial intent injection localize an incomplete governance predicate, and does correcting the predicate close the resulting gap?
**RQ2**  When an AI service fails on real data under a real distribution shift, how much of the resulting *physical* unsafe exposure does each governance mechanism actually remove: admission control, checkpoint rollback, and a context predicate?
**RQ3**  Under what conditions is runtime rollback worth implementing at all, and can that condition be stated as a criterion an engineer can evaluate on their own system before building it?

Section 6.2, Section 8 and Section 9 answer the research questions above; the supporting overhead and scalability characterizations are summarized in Appendix B and provided in full in the artifact. The result is a reproducible systems measurement study, not an industrial deployment validation.

The evaluation combines a controlled testbed with simulated devices and fault injection with a real, publicly available sensing dataset and a real learned model whose own errors, rather than injected faults, drive the AI failure characterization. This paper should therefore be read as a reproducible systems characterization of IoT governance runtime behavior, not as an industrial deployment validation. All scripts, policies, seeds, traces, and analysis code are provided as reproducibility artifacts.

## 2. Related Work

This section positions the work against four directions: governance and policy enforcement mechanisms, the distinction from network security tooling, runtime assurance and recovery in cyber-physical systems, and, most directly, the established literature on automated detection of IoT policy gaps and conflicts.

### 2.1. Governance and Policy Enforcement

Cloud-centric IoT designs introduce latency variability that degrades tail latency performance [10], and centralized coordination with bounded admission control is a standard remedy, but it is applied at the service layer rather than inside the control lifecycle.

Governance frameworks such as the NIST AI Risk Management Framework and ISO/IEC 42001 establish accountability and auditability principles for AI systems [1,2]. However, these frameworks remain descriptive and do not specify mechanisms for embedding machine-checkable policies into runtime environments. The EU AI Act further emphasizes risk management and lifecycle oversight [11], but translating these requirements into runtime-enforceable policy gates remains an open systems problem.

Policy-as-code platforms such as Open Policy Agent (OPA), Kubernetes Gatekeeper, and HashiCorp Sentinel demonstrate that governance rules can be evaluated programmatically at the admission-control layer [12,13,14,15]. These tools are designed for cloud-native deployment admission rather than for IoT control-loop governance, where policy violations can affect cyber-physical behavior. The present work extends policy-as-code evaluation into the IoT runtime lifecycle and empirically compares three backend architectures for policy evaluation overhead.

This work does not propose a new policy-specification language: the gates are simple deterministic predicates, and an equivalent Open Policy Agent (OPA) Rego encoding of G1–G4 is provided in the Supplementary Materials to show the semantics are not tied to our implementation. The motivation for a dedicated runtime is not expressiveness but the IoT control-loop lifecycle that cloud-oriented policy tools do not address: (i) coupling admission to checkpoint-based rollback; (ii) bounded throttling under sensor-driven queue pressure; (iii) hash-linked per-intent audit evidence; and (iv) timestamp-level overhead instrumentation. These lifecycle and measurement capabilities, not the policy language, are the contribution.

### 2.2. Distinction from Security Tools

The runtime is not an intrusion detection, SIEM, or SOAR system. Those tools operate at the network or infrastructure layer and detect malicious traffic or correlate security events; the governance layer operates at the application control layer and gates sensor-originated intents on domain constraints (setpoint bounds, metadata integrity, queue health, audit completeness) before they reach actuators. The two are complementary rather than alternatives.

### 2.3. Automated Detection of IoT Policy Gaps and Conflicts

A substantial body of work automatically detects unsafe states, rule conflicts, and policy gaps in IoT automation. Static analysis and model checking systems such as Soteria [3], IoTSan [4], and HomeGuard [8] enumerate application or rule configurations that reach unsafe states; iRuler [7] applies SMT solving and model checking to find inter-rule vulnerabilities in trigger-action deployments; TAPInspector [9] adds latency and concurrency to the same analysis; IoTGuard [5] and IoTSafe [6] enforce safety policies at runtime, with the latter discovering physical interactions empirically. Methodologically, policy testing rests on an established basis in mutation testing of access control policies [16] and in falsification of temporal logic safety properties for cyber-physical systems [17].

Within this space we contribute an automated, oracle-based policy gap finder specialized to *physical*-actuation safety (Section 6.2): it mutates the declarative gates, generates coverage-guided control intents, and uses an independent physical safety oracle as a metamorphic relation to decide when an admitted intent is unsafe. We do not claim to outperform static analysis systems such as iRuler and HomeGuard on rule interaction depth; the distinguishing result is that policy testing *without* a physical oracle detects none of these physical actuation gaps, whereas the oracle-based finder recovers them automatically. This finder, together with the reproducible closed-loop accounting of governance overhead, rollback cost, audit integrity, and residual AI failure risk, is the contribution.

### 2.4. Runtime Assurance and Recovery in Cyber-Physical Systems

The work most closely related to the rollback component comes from runtime assurance for cyber-physical systems. The Simplex architecture and its descendants combine a potentially untrusted or high-performance controller with a trusted safety controller and a decision mechanism that switches control before the system leaves a recoverable region [18,19]. Restart-based approaches similarly exploit physical inertia to identify time windows within which software recovery can complete without violating a physical safety constraint [20]. Those systems determine whether and when a trusted controller can be engaged; the present study asks the complementary empirical question of how much unsafe physical exposure is actually removed by pre-actuation admission, post-admission rollback and different rejection fallbacks when software decisions are far faster than physical recovery. The sweep of Section 9 identifies a condition analogous to the recoverability requirement in those architectures: rollback materially reduces exposure only when the monitor samples the plant before it can leave the safe set and the plant can be restored before the next unsafe command arrives. This is an empirical operating envelope result for the studied plant family, not a claim of formal equivalence to Simplex or of universal validity.

### 2.5. Positioning

Table 1 locates this work functionally against representative systems; it is a scope-based comparison, not a performance benchmark.

## 3. Governance Runtime Design

### 3.1. System Model and Scope

The target environment is a resource-constrained AI-driven IoT deployment composed of edge devices, lightweight AI services, message-based communication, and a governance plane responsible for policy evaluation and rollback coordination. The governance runtime is not intended to execute on the constrained sensor or actuator endpoints themselves. It is deployed as the governance plane on a more capable node, namely an edge gateway or a dedicated coordination node that mediates between sensor/AI services and actuators; the constrained devices remain pure sources of intents and targets of actuation. This placement is consistent with the reported reference hardware, which represents a gateway class rather than microcontroller-class node, and it is why absolute latencies are reported only to characterize backend overhead and the queue pressure knee, while constrained device measurement is identified as future work. Sensor observations and control intents arrive as timestamped events, each associated with a policy context, service identifier, and device capability descriptor. The system is designed for operational predictability rather than strict determinism. Network delay, queue buildup, service crashes, and temporary replica divergence may occur. The governance plane enforces bounded behavior through admission control, throttling, checkpointing, and rollback.

The failure model assumes crash recovery behavior and intermittent network degradation. Byzantine consensus, malicious key compromise, and arbitrary hardware corruption are outside the current scope. Under network partition, the runtime prioritizes safety and audit consistency over unrestricted availability for state changing intents, following a conservative CP-oriented behavior for governed state transitions. The term “replicated governance state” denotes checkpoint-synchronized metadata under this crash recovery model, not a Byzantine fault-tolerant consensus protocol.

### 3.2. Representative Scenario: Smart Building HVAC Governance

The concrete scenario throughout the paper is a single-zone smart building climate control loop; it makes the governance semantics concrete and is not a physical deployment. Temperature, occupancy and CO_2_ sensors feed an AI inference service that recommends a zone setpoint. Each recommendation is issued as a control intent {device_id, room_id, setpoint, action, timestamp, source, sequence_id} and must pass the governance gates before actuation: G1 requires the setpoint to lie within [15,30] °C and the action to be permitted; G2 requires timestamp, source, and sensor provenance to be present and well formed; G3 throttles non-critical HVAC intents under queue overload; and G4 requires a monotonic sequence identifier and an append to the hash-linked audit trace. If an unsafe setpoint or corrupted metadata is detected, the runtime restores the latest eligible checkpoint, which holds the previously accepted setpoint, the active policy version, the sequence counter, and the audit hash. The characteristic commands, operating conditions, and gate predicates used in the experiments are drawn from this scenario, and Section 6.3 instantiates it with a real learned model on real sensor data.

### 3.3. Architecture

**Figure 1:**
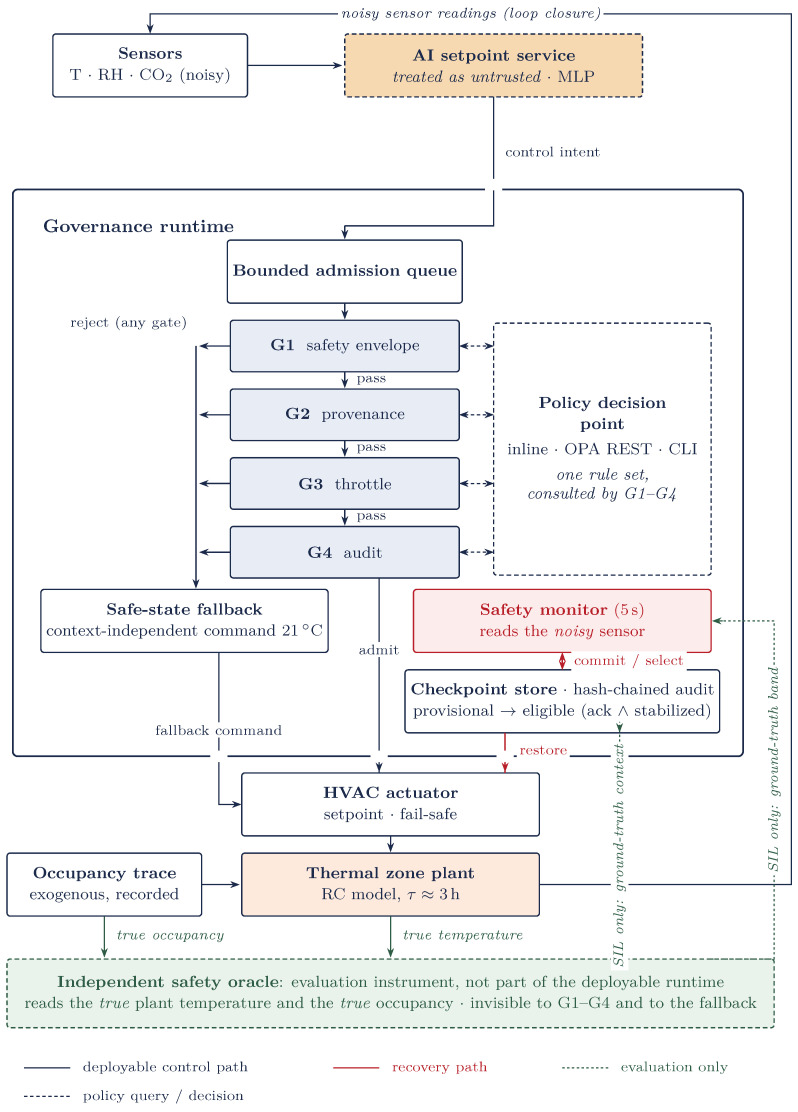
Closed-loop architecture. A control intent from the untrusted learned setpoint service enters a bounded admission queue and must pass gates G1–G4 in order; every gate evaluates the same rule set through the pluggable policy decision point, and a rejection at any gate diverts the intent to the context-independent safe-state fallback. Only an admitted command reaches the actuator and the RC thermal plant. The monitor reads the *noisy* sensor and can restore the latest *eligible* checkpoint (red). The safety oracle (green) reads the *true* temperature and occupancy and belongs to the evaluation testbed, not to the deployable runtime (Section 4.7); the two dotted green links are the recovery path’s evaluation-side dependency, stated in Section 3.6.

The architecture centers on a governance-aware orchestrator responsible for admission control, intent routing, priority scheduling, health monitoring, rollback, and policy enforcement. AI service modules connect through standardized contracts defining input/output schemas, resource constraints, and rollback semantics. A normalized device layer abstracts heterogeneous controllers through protocol adapters unified by a common capability schema, preventing direct module–device interaction and preserving centralized governance enforcement. Figure 1 summarizes the architecture.

End-to-end latency is decomposed as

(1) $$L_{e2e}=L_{\mathrm{queue}}+L_{\mathrm{policy}}+L_{\mathrm{inference}}+L_{\mathrm{device}}+L_{\mathrm{commit}},$$

with tail performance evaluated at the P90 percentile. This decomposition motivates the timestamp-level instrumentation described in Section 4. The orchestrator employs priority queues with O(logQ) scheduling complexity, while policy evaluation and capability lookup operate in constant time.

### 3.4. Governance State Machine

**Figure 2:**
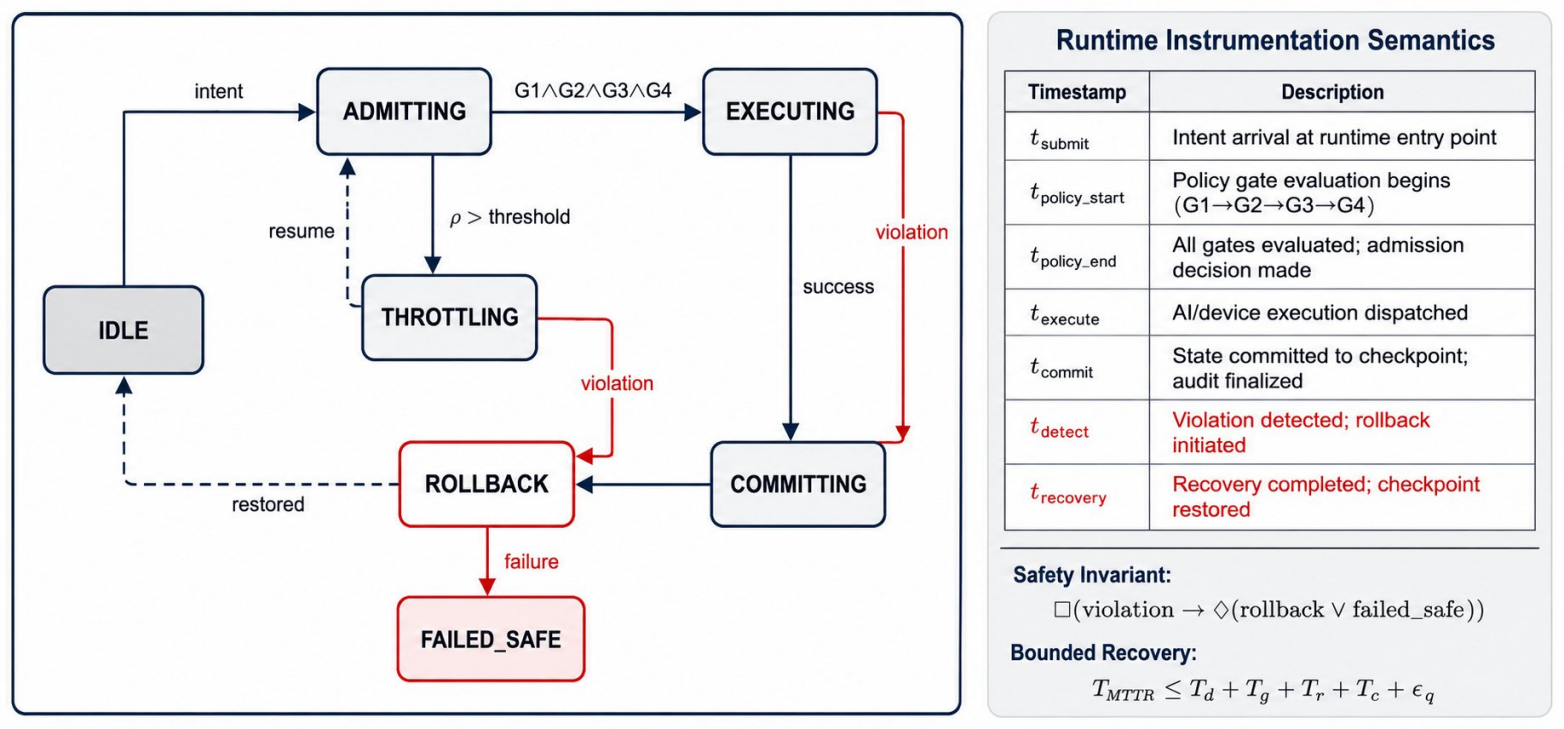
Governance runtime state machine (**left**) with transition guards and instrumentation timestamps (**right**). The safety invariant and bounded recovery property are stated as design invariants.

The governance plane is modeled as a finite state machine to make runtime behavior explicit and reviewable. Figure 2 illustrates the states, transition guards, and associated instrumentation timestamps. Table 2 defines the allowed transitions.

Transitions are triggered by policy outcomes, queue conditions, and runtime health indicators. Table 3 specifies the evaluated attributes, acceptance criteria, and rejection actions for each governance gate.

The temporal recovery property

(2) □(violation→♢(rollback∨failed_safe))

states that every detected violation must eventually lead to rollback or a failed safe state. This is a design invariant, not a formally verified property.

### 3.5. Formal Governance State Model

The runtime is formalized as a state transition system with a rollback operator, a checkpoint set, and an audit log; the full formal model, its admission predicate, and the invariants it guarantees are given in Appendix A.

### 3.6. Checkpoint Semantics and the Scope of Oracle Independence

A checkpoint is a versioned record of a state accepted as a recovery target. We distinguish two stages, and the distinction is load-bearing. A successful software commit creates a *provisional* record; it becomes an *eligible* recovery checkpoint only after the actuator has acknowledged the setpoint and a stabilization predicate holds, namely that the zone lies inside the safety envelope and has settled within 0.5 °C of the commanded setpoint. Rollback restores the latest *eligible* checkpoint, never merely the latest committed command. Without this distinction the mechanism destroys itself: because the plant is inertial it is still in band immediately after an unsafe setpoint is admitted, simply because it has not heated up yet, so committing at that moment would record the offending setpoint as the recovery target and rollback would faithfully restore the fault.

One property of that predicate must be stated precisely. In this software-in-the-loop testbed the envelope test in the eligibility predicate, and the band the runtime monitor uses to declare an excursion, are instantiated with the evaluation testbed’s context-dependent bands, whose occupancy argument is the recorded ground-truth label; the monitor’s temperature argument is the noisy sensor reading, not the true state. The *admission* gates G1–G4 have no access to either quantity, which is what keeps the false negative measurements non-circular. The recovery experiment therefore evaluates rollback under *oracle-assisted context selection* rather than under a deployable context estimator. The idealization was intended to give the recovery path favorable information, but its exact quantitative bias has not been established, because no runtime-visible recovery predicate was evaluated: an estimated context could trigger the monitor earlier or later and could raise or lower measured exposure. The result should accordingly be read as a characterization of the recovery timescale mechanism *under ideal context*: even with ground-truth context available to the recovery predicates, rollback removed only 0.2% of exposure on the evaluated slow plant. It is not a validated bound for deployment. Instantiating these predicates over runtime-visible observables only is the corresponding engineering task, not measured here.

### 3.7. Failure Model and Recovery

Table 4 summarizes the failure conditions and governance responses.

When violations are detected, the governance plane executes a bounded rollback, defined at the design level as (i) suspend pending non-critical intents, (ii) retrieve the latest eligible checkpoint, (iii) restore orchestration state and device-layer settings, (iv) reconcile governance metadata and append hash-linked audit records, and (v) resume admission after queue stability is restored. The design exposes an instrumentation point at each step; the present software evaluation reports the aggregate recovery latency (MTTR) of this path, with per-step decomposition on physical hardware identified as future work.

Governance overhead is decomposed into policy evaluation, audit packaging, checkpoint synchronization, and rollback coordination. This decomposition makes the cost of governance observable and prevents evaluation from treating governance as a zero-cost abstraction.

### 3.8. Governance Admission Loop

The governance admission loop is straightforward: for each intent the runtime records a submit timestamp, evaluates the policy gates in order, dispatches the policy call to the configured backend (inline, HTTP, or subprocess), and then either rejects, throttles, or commits the intent, appending an audit record and a checkpoint on commit. This dispatch point is what enables the backend overhead comparison in Section 6; the full loop is given in the released implementation.

## 4. Evaluation Methodology

**Figure 3:**
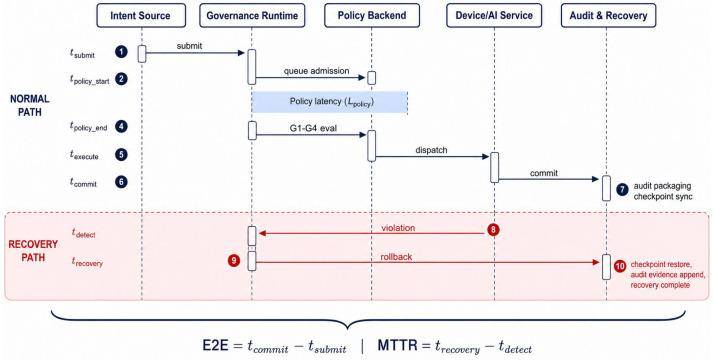
Timestamp-level instrumentation and runtime execution semantics. The normal path (**top**) records queue, policy, execution, and commit timestamps. The recovery path (**bottom**) records detection and restoration timestamps. MTTR is derived directly from recovery timestamps.

The evaluation characterizes the governance runtime through direct timestamp-level instrumentation under controlled testbed conditions. All experiments use software-instrumented execution with simulated devices and controlled fault injection. The results are not presented as industrial deployment measurements. Figure 3 summarizes the instrumentation schema.

### 4.1. Instrumented Runtime Wrapper

Each control step is processed as a governance intent through the full runtime lifecycle. The instrumentation layer records timestamps for every intent: $t_{\mathrm{submit}}$, $t_{\mathrm{policy\_start}}$, $t_{\mathrm{policy\_end}}$, $t_{\mathrm{execute}}$, $t_{\mathrm{commit}}$. For recovery events: $t_{\mathrm{detect}}$, $t_{\mathrm{recovery}}$. Key metrics are derived directly: policy latency as $t_{\mathrm{policy\_end}}-t_{\mathrm{policy\_start}}$, end-to-end latency as $t_{\mathrm{commit}}-t_{\mathrm{submit}}$, and MTTR as $t_{\mathrm{recovery}}-t_{\mathrm{detect}}$.

### 4.2. Policy Evaluation Backends

Three policy evaluation architectures are compared under identical governance logic (gates G1–G4):

*Inline invocation.* Policy predicates are evaluated as direct function calls within the runtime process, serving as the latency baseline.

*HTTP REST endpoint.* A standalone HTTP server evaluates policy predicates via a REST API on the loopback interface. Each intent triggers a JSON-serialized POST request and response, representative of architectures where the policy engine runs as a separate service (e.g., OPA in HTTP mode).

*Subprocess CLI execution.* Each intent triggers a separate process invocation that reads the intent from standard input and writes the policy decision to standard output, representative of CLI-based policy tools (e.g., OPA eval).

All three backends evaluate the same four governance gates, ensuring that observed overhead differences reflect only the evaluation architecture.

### 4.3. Workload Model and Fault Injection

The workload consists of control intents generated for a configurable number of simulated devices (10–5000). The AI inference service is abstracted as an intent source in this tier: the runtime acts only on the emitted intent, so substituting a specific model would not change the governance decision for a given intent, and direct generation gives controlled coverage of malformed inputs a trained model could not guarantee. Section 8 and Section 8.5 replace the abstraction with trained and deterministic controllers driving the plant. Seven fault types are configured (out-of-range and boundary setpoints, null timestamp, null source, queue flood, and two combined cases), and the artifact lists them and the generator parameters in full. The recall battery of Section 6.2 uses the six that open a physical actuation gap; queue flood is excluded, since it degrades throughput without changing the setpoint.

The ground-truth unsafe classification is deliberately broader than the policy gates: an intent is considered unsafe if its setpoint is outside range, its action is invalid, or its metadata source is null. This asymmetry between policy scope and ground truth enables systematic detection of policy gaps.

### 4.4. Experimental Configurations

Two Tier-1 configurations are reported here, each with 30 independent random seeds: backend comparison (three policy backends at 200 devices, Section 6.1) and scalability (10 to 5000 devices, Appendix B). The artifact additionally runs an ablation, a cloud-style baseline comparison, a network sensitivity sweep and an adversarial injection battery; none supports a claim made here, so they are reported in the artifact only.

### 4.5. Statistical Analysis

Across seeds, medians with bootstrapped 95% confidence intervals (10,000 resamples) are reported [22]. Pairwise comparisons use the Wilcoxon signed-rank test [23] (paired by seed) with Cliff’s delta as a non-parametric effect-size measure [24,25]. Effect sizes are interpreted as negligible (|δ|<0.147), small (<0.33), medium (<0.474), or large (≥0.474). Significance is assessed at α=0.05.

**Experimental unit, independence, and exclusions.** The experimental unit is stated with each result. In the simulation and closed-loop tiers it is an independent random seed: 30 seeds for the backend and scalability experiments, and, for the closed-loop and robustness runs, a seed that draws a fresh intent stream and, in the SIL tier, a fresh sensor noise realization. The reported intervals are therefore taken across seeds. In the real-stack prototype (Section 7), the 240 published control intents are observations within a single service run, not 240 independent experiments; we therefore report them as counts (admitted/rejected, actuator applications, and audit chain integrity) and attach no seed-level interval to them. Runs that fail to complete, for example because a broker does not start, are discarded and re-run rather than partially scored; no missing value imputation is applied, and no point is removed from any reported distribution as an outlier. We make no statistical-power claim. The software environment in which the reported results were produced is given in Table 5.

### 4.6. Reproducibility

The governance overhead characterization (Appendix B) was obtained using a reference workstation (Intel Core i7-12700, 32 GB DDR5, Ubuntu 22.04 LTS), with the HTTP REST and subprocess CLI backends evaluated through a deterministic, seed-controlled timing model calibrated to the loopback request and process instantiation overheads observed on that host, so that backend-level architectural overhead is isolated from operating system scheduling jitter. The closed-loop study (Section 8), the plant sweep (Section 9), the robustness audit, and the Tier-3 prototype (Section 7) were executed live on a Windows 10 workstation (Python 3.10.20), whose host-stamped run transcripts are included in the artifact, and were reproduced independently on a Linux host (Python 3.10). Every reported value is regenerated by the released code from a fixed seed, and each run writes a host-stamped transcript so that any reproduction leaves an auditable record.

The complete experiment infrastructure is provided as a reproducibility artifact [27,28]: runtime wrapper source, policy backend implementations (inline, HTTP server, CLI evaluator), Rego policy definition, experiment runner with all six configurations, statistical analysis scripts, and generated CSV traces. All experiments are reproducible given the same random seed and configuration.

### 4.7. Independent Physical Safety Oracle

Closed-loop experiments are scored by an independent physical safety oracle: a deterministic function that observes the true zone temperature and true occupancy, neither of which is available to the deployed governance gate. It defines the context-dependent safe set as S(o)=[20,25] °C when the zone is occupied and [15,30] °C when it is vacant; the occupied band is a pragmatic office comfort range informed by thermal comfort guidance [29], not a complete ASHRAE 55 compliance calculation, which would additionally require operative temperature, humidity, air speed, clothing and metabolic rate. Section 10 varies both bands to test whether the findings depend on the thresholds.

For true zone temperature T(t) the instantaneous excursion e(t) is $T_{\min}\left(o_{t}\right)-T\left(t\right)$ below the band, zero inside it, and $T\left(t\right)-T_{\max}\left(o_{t}\right)$ above it; unsafe physical exposure is the time integral E=∫e(t)dt, reported in °C·min. For 30 min after an occupancy transition the oracle applies the union of the outgoing and incoming bands, which avoids charging the controller for a ramp the modeled plant cannot complete instantaneously; strict scoring without the grace period is reported in Section 10.

The oracle is structurally independent of the *admission* policy: the gates never receive it, it observes variables unavailable to them, and it cannot alter any admission decision, so an admitted action classified as unsafe by the oracle is an oracle-defined false negative rather than a restatement of the gate predicate. It is not independent of the *recovery* path in this testbed, where checkpoint eligibility and the runtime monitor select their band from the same ground-truth context; Section 3.6 states what that does and does not license. The oracle remains an author-defined instrument of the evaluation, not a component of the deployable runtime.

## 5. Evaluation Ladder

Cyber-physical evaluation is conventionally staged, and being explicit about which rung a result sits on is the difference between a scoped claim and an overclaim. We therefore state the ladder first, place every result on it, and name the rung we have not climbed. Table 6 states the four rungs and what each one requires.

Three points about this ladder are load-bearing.

We present the rungs in the order Tier 1, Tier 3, Tier 2. This is deliberate. The central argument of Tier 2 turns on the *ratio* between what a governance decision costs and what the physical world costs, and that ratio is meaningless unless the numerator is a measured number rather than a modeled one. So we establish the measured decision cost on the real stack first, and only then contrast it with the physical consequence.

**The controller is the same code at every rung.** What changes as one climbs is the environment, not the governance implementation. That is enforced structurally by a hardware abstraction layer (Section 5) with a single interface and interchangeable backends, and a mechanical parity check verifies that the sensor and actuator backends for simulation and for the Raspberry Pi implement it identically. The claim that the hardware evaluation is an interface swap rather than a rewrite is therefore checkable rather than asserted.

**Tier 2 is SIL, not HIL.** The distinction matters and we make it explicitly, because the two are routinely conflated. In a hardware-in-the-loop design, the real actuator and sensor hardware are in the loop and the plant is simulated in real time. In a software-in-the-loop design, the real controller *software* runs against a plant *model*. Our closed loop is the latter: real governance code, real recorded sensor data, a real learned model, and a simulated room. **No physical hardware was operated in this study.** Every physical quantity we report is a plant model quantity, and Section 9 is the direct response to the objection that our conclusions might therefore be artifacts of that model.

**The missing rung is named, not hidden.** Tier 4 requires a Raspberry Pi with an SCD40 CO_2_ sensor, a BME280, an opto-isolated relay and a servo. The backend for it is written, released, and parity-checked; it has not been run. It would supply three quantities this paper does not have: the physical actuation latency, the power consumption, and a demonstration of the CO_2_ context predicate on a live sensor. The criterion of Section 9 yields a testable, falsifiable prediction about which rollback benefit regime a Tier 4 experiment would fall in. It does not predict everything Tier 4 would reveal: live sensor noise, actuator dynamics, relay and servo behavior, power draw, and operating system jitter may all differ from their modeled counterparts, and surfacing those is precisely the purpose of that rung.

### The Hardware Abstraction Layer

A single interface separates the governance runtime from its environment. SensorHAL exposes read(); ActuatorHAL exposes apply_setpoint() and enter_failsafe(). Two backend pairs implement it: a simulation pair, driving the plant model of Section 8.1, and a Raspberry Pi pair issuing real I^2^C reads (SCD40, BME280) and real GPIO writes (relay on GPIO17, servo on GPIO18). The Pi actuator returns the *measured* physical actuation latency, which is exactly the quantity this paper declares it does not have; that sentence changes when the backend is run, and not before.

Everything above the interface, namely the AI service, the policy gates, the checkpoint and rollback logic, the safety monitor, and the audit chain, is identical across backends. A parity check enumerates the interface’s abstract methods and verifies that all four backends implement them concretely; it is part of the released test suite (25 tests), so a reader can confirm the claim rather than take it.

## 6. Tier 1: Simulation

All values are medians over 30 independent seeds with bootstrapped 95% confidence intervals. Statistical comparisons use the Wilcoxon signed-rank test with Cliff’s delta.

### 6.1. Policy Backend Overhead

Table 7 reports runtime metrics across three policy evaluation backends at 200 simulated devices.

The inline backend evaluates a policy in about 1 μs; the HTTP REST backend adds about 1 ms (JSON serialization and loopback socket overhead) and the subprocess backend about 26 ms (per-intent process creation). All three admit identically, so the differences reflect the invocation architecture rather than the policy logic (Wilcoxon $p<10^{-9}$, Cliff’s δ=−1.0 on policy P90; recovery time is unaffected, |δ|<0.22). The ordering is expected and not claimed as novel; what the timestamp-level decomposition establishes is that governance overhead is dominated by queue contention rather than policy evaluation beyond roughly 1000 devices, which moves the engineering lever from “optimize the policy backend” to “manage admission and queueing”. These figures characterize a gateway class node and are not extrapolated to microcontroller-class endpoints.

### 6.2. Automated Policy Gap Discovery

A recurring objection to hand-picked fault injection is that omitting one predicate by hand trivially yields *some* unsafe admission rate whose magnitude is an artifact of the chosen omission. We therefore replace the single hand-injected omission with an *automated* policy gap finder that reports what it discovers without being told where to look. It combines three ingredients: *mutation operators* over the declarative gates (drop or weaken each predicate); *coverage-guided, property-based generation* of control intents that exercise every gate decision boundary and every latent physical zone; and an *independent physical safety oracle* used as a *metamorphic relation*: the gate never sees the per-zone true safe band, so an “unsafe-and-admitted” intent is a genuine physical gap rather than a restatement of the gate’s own predicate.

**Figure 4:**
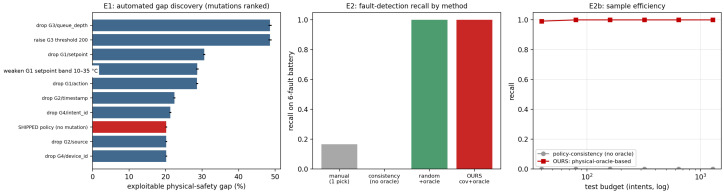
Automated policy gap discovery. (**Left**): exploitable physical safety gap opened by each single-predicate mutation, ranked; the shipped policy’s own gap (red) is surfaced automatically. (**Centre**): fault detection recall by method; only oracle-based search finds physical actuation gaps. (**Right**): recall versus test budget; without the physical oracle, recall is zero at every budget.

Ranking every single-predicate mutation by the physical safety gap it opens (Figure 4, left; 30 seeds, bootstrap 95% confidence intervals) yields an interpretable ordering: dropping the queue-health check (G3) is worst at 48.6% [48.2, 49.0], the setpoint bound (G1) next at 30.6%. Decisively, the same ranking flags the *shipped* policy itself at a 20.2% [19.9, 20.5] exploitable gap, surfacing the missing source-provenance predicate automatically rather than by a human’s guess. Enabling that G2 predicate closes the shipped-policy gap (Appendix C), confirming a policy-specification omission rather than an architectural limit.

The load-bearing ingredient is the physical oracle. On a battery of six injected faults (Table 8), a human testing one hand-picked predicate recovers 1/6 and policy-consistency testing *without* a physical oracle recovers *none* at any budget, because a rule-level check cannot know that an admitted intent is physically unsafe; the oracle-based finder recovers all six from a budget of forty intents, in 0.11 s. Random intent generation with the same oracle also reaches full recall on this battery (Table 8), so the decisive ingredient is the oracle, not the search strategy: coverage-guided generation guarantees that every decision boundary and latent zone is exercised at a fixed budget, but on this battery it confers no measured advantage. The novelty is therefore not a new solver but the metamorphic use of an independent physical safety oracle to turn policy testing into *physical*-gap discovery. We do not claim to outperform static analysis systems such as iRuler [7] or HomeGuard [8], which reason over rule interactions we do not model; the finder answers the complementary question of how much physical exposure a policy omission admits.

### 6.3. Real AI Service on Real Sensor Data

To ground the AI failure characterization in a realistic AI-driven IoT setting rather than synthetic fault injection, we replace the simulated inference service with a real learned model trained on a real, publicly available occupancy-sensing dataset [26]: measurements of temperature, humidity, CO_2_, and humidity ratio from an instrumented office. The light channel is excluded, because it trivially reveals occupancy and is frequently absent in HVAC deployments. The dataset provides two evaluation periods recorded at different times, which yields a *real* distribution shift; no faults are injected by hand anywhere in this experiment.

**Model and training.** The inference service is a multilayer perceptron regressor with two hidden layers of 32 and 16 ReLU units (705 learned parameters), trained with the Adam optimizer under a fixed seed. It is fitted on the 8143 rows of the UCI training split (SHA-256 034506…631f; 1729 occupied, 6414 vacant) using the four standardized features above and converges in 327 iterations (of a 600 cap) with the loss falling from 178.7 to 0.34. It attains a training mean-absolute setpoint error of 0.40 °C and, read as an occupancy classifier at the 19.5 °C midpoint, recovers occupancy with 96.4% accuracy. This does not hold off-distribution: the mean-absolute error rises from 0.40 °C on the training split to 1.69 °C on the held-out datatest split and 3.32 °C on the shifted datatest2 split (Table 9). This degradation is not a defect to be tuned away: it is the realistic behavior that gives the governance layer something to catch. The specification is fixed and seed-controlled, so the entire training reproduces bit-for-bit; the released full_training.py prints every stage, from the data fingerprint through each iteration of the loss. Reproduction is not confined to the pinned environment: re-executing the released pipeline at release v1.2.0 on an independent workstation running Windows 11 with Python 3.13, NumPy 2.4.6, pandas 3.0.3 and scikit-learn 1.9.0 returned 127 of 127 reported values matching, including all 33 cells of the plant sweep and the 18 robustness perturbations. This model’s dependence on architecture and seed is stress-tested in Section 10 (six learners including a linear one, four seeds).

Following the smart building HVAC scenario of Section 3.2, a multilayer perceptron regressor maps live sensor readings to a recommended setpoint, with a control target of 22 °C when the room is occupied and a 17 °C energy setback when it is vacant. The model’s own errors, namely miscalibration and extrapolation under shift, generate the control intents. The context-blind gate G1 admits an intent if the commanded setpoint lies in [15,30]; it never observes true occupancy. An independent physical oracle uses the true occupancy label: a command is physically unsafe if it falls outside [20,25] when the room is occupied, or outside [15,30] when it is vacant. A corrected gate adds a single predicate on CO_2_, a context signal already carried in the intent, rejecting an energy-setback command when CO_2_ indicates the room is in fact occupied.

**Figure 5:**
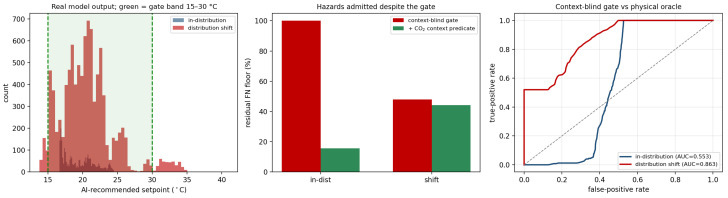
Real learned model on real sensor data. (**Left**): distribution of AI-recommended setpoints; the shaded region is the gate’s admissible band. (**Centre**): residual hazards admitted despite the gate, with and without the CO_2_ context predicate. (**Right**): the context-blind gate scored against the independent physical oracle. In the left panel the in-distribution and shifted histograms are overlaid semi-transparently; where they overlap the blue in-distribution histogram is covered by the red shifted one and the overlap reads as dark reddish brown.

Table 10 and Figure 5 report the outcome. In distribution the model errs subtly: every recommended setpoint remains inside the gate’s band, so the context-blind gate admits *all* physically unsafe commands and its discrimination is close to chance (AUC 0.553). Under real distribution shift the model extrapolates far out of range, so a fraction of commands leave the global band and the gate does catch those (AUC 0.863), yet a large residual of in-band, context-unsafe commands still passes. Adding the CO_2_ predicate reduces the residual from 100% to 15.7% in distribution at a false positive cost of 2.7%, but it barely helps under shift (48.0% to 44.2%), because the context signal itself shifts. Context-aware governance therefore helps and is itself distribution-shift-sensitive.

That a fixed, context-blind filter cannot capture a context-dependent safe set is established in principle by responsibility-sensitive safety [30] and by reachability-based shielding [31]. Our contribution here is narrower and empirical: within the testbed we quantify the resulting false negative floor for a deterministic admission gate and show that it is a structural property of the gate’s observable predicate space rather than a tuning artifact. Rollback recovers *detected* faults; the residual hazards characterized here are, by construction, not detected at admission time, so the tamper-evident audit trace is what records them and keeps them attributable for post-hoc analysis. Closing this residual requires context-aware admission, which we treat as future work.

### 6.4. Scalability and Queue Pressure Analysis

This analysis uses the M/M/1 queue pressure approximation defined in Appendix B.

Table 11 reports P90 latency across device counts from 10 to 5000. The knee location is a property of the calibrated service and arrival parameters, not a universal constant: a faster policy backend or a higher per-node service rate shifts it to a larger device count, and a slower one shifts it earlier. The qualitative conclusion, that queue contention rather than policy evaluation is the binding constraint at scale, is invariant to these choices, because policy evaluation remains orders of magnitude below transport and queueing cost across the measured range (Appendix B).

Policy evaluation latency remains constant across all device counts (0.001 ms P90). End-to-end latency remains below 10 ms up to 500 devices, then increases sharply to 79.8 ms at 1000 devices due to queue pressure saturation. Beyond 2000 devices, latency plateaus near the M/M/1 saturation cap of approximately 105 ms. These thresholds are specific to the calibrated queue parameters (r=1 intents/s, $\mu_{0}=1520$ intents/s, α=0.0005); different workload profiles or physical network conditions would shift the knee location. MTTR remains stable at approximately 1.3 ms, indicating that recovery bypasses the admission queue. This separation implies that queue-aware admission control should be the primary optimization target for scaling, while policy evaluation optimization would yield negligible benefit.

## 7. Tier 3 (Prototype): The Same Governance Plane on a Real Stack

Tier 1 models the timing of the out-of-process policy backends so that the comparison is reproducible across machines. Tier 3 measures the governance path directly rather than modeling its timing. The same governance code runs against a real MQTT broker (a real publish/subscribe network hop), an in-process executable policy decision point evaluating the same G1–G4 gate predicates, and a real append-only hash-chained audit log on disk; every number in this section is measured on that stack. An equivalent Open Policy Agent (OPA) Rego encoding of the gates, together with a docker compose deployment that runs OPA as an external HTTP decision point, is provided and conformance-tested; the latencies reported here are the in-process decision path, and the out-of-process cost of the same gates through a real OPA server over HTTP is measured directly (median 0.94 ms; Table 12). These are service-run measurements of one machine each and they move with the host: an independent reproduction on a second workstation returned 0.447 ms median and 0.593 ms P90 on the MQTT path and 0.666 ms median through OPA, with 240/240 decision agreement in both cases. The argument rests on the order of magnitude, sub-millisecond decisions against a 61 min physical recovery, and not on any single value.

The governance plane also runs as a deployable MQTT service: AI services publish control intents to intents/<device>, the plane evaluates G1–G4 through a pluggable policy decision point, admitted commands are published to actuators/<device>/cmd, and every decision is appended to a disk-backed hash-chained audit log. Implementation details (topic layout, the OPA Rego encoding and its conformance set, the simulator and Raspberry Pi actuator adapters, and the docker compose deployment) are in the artifact.

Nothing in the measurement path is modeled: a real broker, network hop, policy call and disk write. Table 12 reports 240 real control intents traversing the stack, comprising safe commands, out-of-band setpoints, disallowed actions, stale metadata, and forged provenance. The plane admitted 120 and rejected 120, the actuator applied exactly the 120 admitted commands, and the persistent audit chain verified intact; modifying a single record on disk caused verification to fail, confirming tamper evidence on the persisted log rather than only in memory.

Two properties of this measurement matter. First, the governance decision latency is *measured*, not assigned: median 0.44 ms and P90 0.64 ms on a Windows workstation, and median 0.09 ms and P90 0.34 ms on a Linux host. The spread across hosts is expected and is reported rather than hidden; the point is that both are real measurements of the same released service, independently reproduced on two operating systems. Second, the policy gap diagnosis of Section 6.2 reproduces *live* on the running stack rather than in offline analysis: under the shipped policy all 40 forged-provenance intents were admitted, and under the corrected policy, which differs by one predicate in a configuration file, all 40 were rejected.

We are explicit about what this does and does not establish. It establishes that the governance mechanism runs as a real service on a real message bus with a real policy engine and persistent audit, and that its decision cost is small and measurable. It does not establish deployment on constrained endpoints, physical actuation timing, or production-scale operation. In particular, the broker, the policy decision point, and the audit log are co-located on a single host and communicate over the loopback interface, so these numbers measure the real software stack (message bus, policy engine, and disk) but not wide-area network transit; a distributed deployment would add transport latency we do not claim to characterize here. The Raspberry Pi GPIO adapter is released to enable the hardware evaluation, and we do not report hardware numbers we have not taken.

The same gates were additionally evaluated through a real Open Policy Agent server (v1.18.2) over the loopback HTTP interface, with the Rego encoding uploaded to opa run –server and queried once per intent. On the Windows host, over 240 intents with every OPA decision verified against the runtime’s, the decision costs a median of 0.94 ms (P90 1.05 ms): the out-of-process policy path is roughly twice the in-process cost and consistent with the Tier-1 HTTP backend yet remains more than six orders of magnitude below the 61 min physical recovery. It is reproduced by python experiments/opa_http_latency.py against a running OPA server.

This is the rung at which the paper’s latency claims are made. When Section 8 contrasts a 0.44 ms governance decision with a 61 min physical recovery, the 0.44 ms is this measurement and not a model. The contrast is between a measured software cost and a modeled physical one, and we state which is which because the entire argument of Section 9 relies on their ratio.

## 8. Tier 2 (SIL): Closing the Loop Through a Physical Plant

The experiments above measure the governance layer in software. They cannot, by construction, distinguish a rejected intent from an admitted-then-rolled-back one, because software state has no inertia: both end with the state restored. A room does not. Once a bad setpoint has been admitted, the room begins to heat, and rolling the setpoint back does not roll the heat back. This section closes the loop through a physical model so that the difference becomes measurable, and it is where the paper’s principal result is obtained.

### 8.1. The Plant

We model a single-zone office as a lumped-parameter (RC) thermal system driven by an HVAC actuator:

(3) $$C\;\frac{dT}{dt}\;=\;\frac{T_{\mathrm{out}}-T}{R}\;+\;P_{\mathrm{hvac}}\;u\;+\;Q_{\mathrm{int}},\;u\in \left[-1,+1\right],$$

with $C=1.2\times 10^{6}$ J/K, R=0.009 K/W (so τ=RC≈3 h), $P_{\mathrm{hvac}}=3$ kW, $T_{\mathrm{out}}=12$ °C, and internal gains $Q_{\mathrm{int}}=200+100\;n$ W for *n* occupants. These are order-of-magnitude values for a small office zone, stated in full so they can be challenged. A local PI loop inside the actuator drives u∈[−1,1] toward the governed setpoint, with the integrator held whenever the command saturates (proportional gain fixed at $k_{p}\;=\;0.8$, so errors above ∼1.25 °C saturate the actuator; integral gain scaled with capacitance, $k_{i}=0.003\;C/C_{\mathrm{ref}}$ with $C_{\mathrm{ref}}=1.2\times 10^{6}$ J/K); the state is integrated by forward Euler with $\Delta t=\max\{0.05\;s,\;\min\left(5\;s,\;\tau /50,\;T_{\mathrm{mon}}/2\right)\}$, which is 2.5 s at the default monitor period $T_{\mathrm{mon}}=5$ s; each run initializes the zone at the first recorded temperature of its trace; and the AI service issues one setpoint per minute, the dataset cadence. Two of their consequences every result in this section: the maximum heating rate is +0.109 °C/min and the maximum cooling rate is −0.207 °C/min. Excursions therefore take tens of minutes to build and minutes to clear, and that slowness is the finding.

This is a digital twin, not a room. **No physical hardware was operated in this study.** Every physical quantity reported below is a twin quantity. Section 9 addresses the obvious objection, that the conclusions might be artifacts of these particular parameters, by sweeping them.

### 8.2. The Independent Safety Oracle

Every arm is scored by the independent oracle defined in Section 4.7. One of its provisions is specific to this plant. When occupancy changes, the band the room must satisfy changes instantly, but the room cannot. A zone at the 17 °C vacant setback must climb 3 °C to re-enter the occupied band, which at the plant’s maximum heating rate requires ≈ 28 min of full-power heating. No controller of any kind, governed or clairvoyant, can beat that. Without a grace period the metric would mix governance-attributable exposure with the unavoidable thermal ramp that the 22/17 °C setback strategy itself creates; strict scoring without the window is reported separately in Section 10. The oracle therefore applies, for a 30 min grace window after each occupancy transition, the union of the outgoing and incoming bands. The window still flags every genuine hazard while forgiving the compulsory ramp, and Section 10 validates it: a *perfect* model handed ground-truth occupancy scores exactly 0.0, while the ungoverned arm still scores 1185.3 °C·min through the same window.

### 8.3. Experimental Arms

The AI service is the same MLP setpoint regressor of Section 6.3, trained on datatraining.txt and driven by the recorded sensor stream. Three policies differ from one another by *one dictionary entry*, not by a code change, which is what makes the comparison honest:

- **Shipped:** bounds the setpoint to the equipment range [15,30] °C and checks metadata. It is a transparent, deterministic baseline consisting of equipment bounds and metadata checks: valid at the equipment level but blind to occupancy context.
- **Corrected:** shipped, plus one conditional predicate: if CO_2_ exceeds the 95th percentile of vacant *training* CO_2_ (755 ppm), the setpoint must lie in the occupied band. The threshold is derived from the training split only, never from the evaluation splits.
- **Oracle:** the same predicate conditioned on *true* occupancy. It is not deployable, since knowing the occupancy would obviate the model, but it bounds what any admission gate could achieve and so separates the runtime’s contribution from the policy’s.

### 8.4. What Each Governance Mechanism Actually Buys

**Figure 6:**
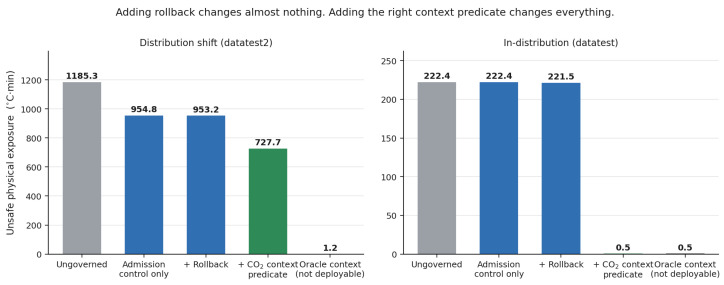
Unsafe physical exposure under each governance layer, in and out of distribution. Adding rollback changes almost nothing; adding the right context predicate changes everything. Values are those of Table 13.

Table 13 gives the result, and Figure 6 plots it. The third and fourth rows must be read together.

**Rollback recovers almost nothing.** Admission control cuts exposure from 1185.3 to 954.8. Adding checkpoint rollback takes it to 953.2, an improvement of 0.2% of the exposure admission control left (0.1% of the ungoverned total), and the peak excursion does not fall at all (3.75→3.81 °C). This is not a defect of our implementation. It is a consequence of the timescales, and Table 14 makes the mechanism explicit: the governance *decision* takes 0.44 ms on the real MQTT stack of Section 7, whereas *physical recovery* of the plant takes a median of 61 min and up to 146. By the time the runtime has restored a safe setpoint, the heat is already in the room. Rollback *ends* an excursion; it does not undo one. Reporting only the decision latency describes the cheap half of rollback and silently omits the expensive half.

**Figure 7:**
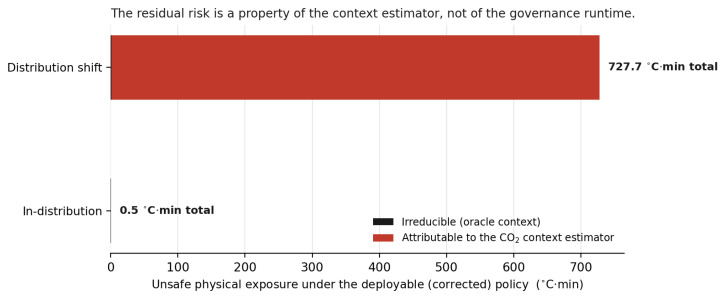
Where the residual physical risk lives. Under the deployable (corrected) policy, the portion attributable to the CO_2_ context estimator is separated from the irreducible portion an oracle context would also incur. Values are those of Table 13 and its in-distribution counterpart.

**Prevention is what works.** The oracle policy, using the same runtime, the same rollback and the same plant, and differing only by one predicate that happens to be right, lands at 1.2 °C·min. The runtime mechanisms are therefore not the limiting factor. The policy’s estimate of context is. The deployable proxy for that context sits between the two extremes: the corrected CO_2_ predicate leaves 727.7 °C·min, because under distribution shift CO_2_ stops tracking occupancy. That gap, 727.7 against 1.2, is a direct measurement of how much residual *physical* risk is attributable to the context estimator rather than to the governance runtime (Figure 7).

In distribution the picture is quite different: the corrected predicate reduces exposure from 222.4 to 0.5 °C·min, matching the oracle exactly. Table 15 reports 221.5 for the same controller: that is the separate controller-baseline run, in which no gate rejection occurs on this trace, so the two are distinct measurements. The gate did not change; the world did.

### 8.5. What the Learned Controller Buys, and What It Costs

The study governs a learned controller, and it is fair to ask what that controller contributes beyond a deterministic rule and what risk it introduces in exchange. We answer by measurement and state the unit of comparison first: what is compared is a *governed system*, not a controller in isolation, because the gate intervenes far more often on the learned controller’s output than on the rule’s. Three setpoint controllers run through the *identical* governed loop (shipped gate, rollback, safe-state fallback) on the same recorded traces and are scored by the same oracle: the paper’s four-feature MLP; a deterministic single-rule thermostat with the same information provenance as the corrected gate (CO_2_ above the training vacant 95th percentile → occupied setpoint, else setback), representing a transparent single-signal baseline implementable without machine learning; and a constant 22 °C controller that never sets back. The heating demand proxy integrates the steady-state power required to *hold* each commanded setpoint, $\max\{0,\;\left(s_{t}-T_{\mathrm{out}}\right)/R-{\dot{Q}}_{\mathrm{int}}\}$ summed per minute. It is computed from commanded setpoints, not from the actuator duty cycle $P_{\mathrm{hvac}}u\left(t\right)$, so it omits transient dynamics and actuator saturation and is reported in kWh-equivalent units for comparability only; it is not an energy measurement.

Table 15 makes three points. First, under the evaluated shift the governed MLP system incurs less unsafe exposure than the governed single-rule system: in distribution the rule is as safe, but under shift its exposure is 50% higher (1427.7 against 953.2), because it inherits the fragility of its single context signal. This is one comparison, on one plant, under one recorded shift, and it does not establish a general robustness advantage of learned control over richer deterministic controllers; the MLP also carries a 38% higher heating demand proxy (115.6 against 83.6), so the result is a safety–demand trade-off that bounds the question rather than settling it. Second, setback control of any kind is what creates the safety problem: the constant controller incurs zero avoidable exposure at a 25–73% higher modeled demand, so the risk the governance layer manages is the price of the energy saving, not an artifact of the AI. Third, governance interacts *more* with the learned controller: under shift the gate rejects 306 of its extrapolated commands and none of the rule’s, which are in-band by construction. We therefore do not claim a learned controller is *necessary* here, only that a setback controller of some kind creates the hazard the governance layer manages and that a learned one is a plausible instance whose failure modes admission control exists to catch; cost, maintenance, interpretability and certification are not measured. The MLP configuration was fixed before the comparison and not optimized on either evaluation split, and Section 10 shows the conclusions hold across six learners.

### 8.6. Rejection Semantics Dominate Rejection Accuracy

A gate that only vetoes is not a controller. When an intent is rejected, the actuator keeps tracking whatever setpoint it already held, and if the context has changed, that setpoint is now wrong too. What the runtime *does* when it says no is therefore a design decision, and Table 16 shows that it matters more than how accurately the gate says it.

**Figure 8:**
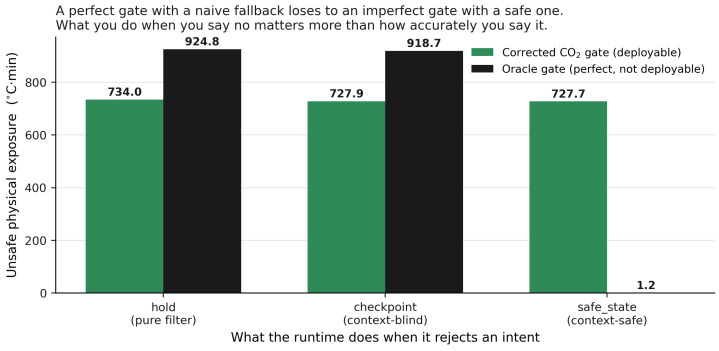
Rejection semantics against rejection accuracy. Values are those of Table 16.

An oracle-accurate gate that reverts to a context-blind state leaves 924.8 °C·min. A substantially less accurate gate that reverts to a context-independent safe state leaves 727.7. The perfect gate *with* the safe fallback leaves 1.2. The fallback dominates. The reason a checkpoint is not enough is instructive: a 17 °C setback that was verified safe while the room was *empty* is not safe once somebody walks in, so reverting to it reverts to a different wrong answer. A context-independent safe state is safe precisely because it does not need the context the runtime does not have. This is invisible in a software-only testbed, where restoring a variable is instantaneous and all three fallbacks are indistinguishable (Figure 8).

### 8.7. Where Rollback Is Not Available at All

Some actuations cannot be undone by issuing another command: a compressor lockout, a fired suppression system, a purged tank. We model an actuator that latches above 28 °C and ignores every subsequent command. Under the shipped policy the runtime loses authority and enters FAILED_SAFE at minute 1050, having accrued 4951.7 °C·min, with availability (the fraction of the run in which the runtime still holds actuation authority) reduced to 10.8%. It does not claim to have recovered, because it has not: the room is latched hot and only an operator can clear it. We regard the explicit separation of “the runtime relinquished authority” from “the runtime lost authority” as a necessary property rather than a detail. Under the corrected policy the offending intent is never admitted, so the irreversible actuator is never reached. Against irreversibility, prevention is the only thing that works, which is an argument for policy correctness rather than for better rollback.

### 8.8. Governance Cost

Gate evaluation costs 3.5 μs (median, shipped) and 4.0 μs (corrected) on the Linux host; the same measurement on the Windows host gives 4.5 and 5.2 μs. We report both, because a microbenchmark at this scale is as much a property of the machine as of the code. The audit chain verified intact across 9761 entries on both. What is invariant, and what the argument actually rests on, is the *difference*: the context predicate costs well under a microsecond per intent on either host (+0.5 to +0.7 μs across repeated runs) and removes 226 °C·min of unsafe exposure. That ratio, and not the absolute latency, is the deployment argument, and it is the exact inverse of the ratio rollback offers.

### 8.9. A Mechanism That Never Fires

We implemented a rollback budget, intended to stop the runtime from undoing the same mistake indefinitely: if it is rolling back every few minutes, the model is systematically wrong and the honest response is to hand the zone to the fallback thermostat and call an operator. We then checked whether it ever fires. It does not: the shipped policy triggers only 9 rollbacks in 163 h, never three within any one hour, so no budget in the range we tested is ever reached, and every setting gives an identical result. We report this rather than dropping it, because a mechanism that never fires is not a contribution and we do not present it as one, and because it means the only condition under which this runtime legitimately reaches FAILED_SAFE in our workload is irreversible actuation.

## 9. When Is Runtime Rollback Worth Having?

The result above is true of *our* plant, and the obvious objection is that a room with a three-hour time constant is not a valve, a fan, or a robot arm. If physical recovery took seconds rather than an hour, rollback would surely work. Stated as a fact about governance, our result would then be wrong; it is a fact about governance *on a slow plant*. Rather than defend one set of parameters we cannot validate against a real building, we sweep them and let the sweep draw the boundary. This turns the study’s largest vulnerability into its most transferable output: a criterion an engineer can evaluate on their own system before building a mechanism that will not help them.

### 9.1. Method

We scale the zone’s thermal capacitance *C*. This is the honest single knob, because it scales exactly the two quantities that matter, the recovery time constant (τ=RC∝C) and the maximum slew rate (∝1/C), while leaving the hazard geometry alone: the equilibrium temperature $T_{\mathrm{out}}+R\;Q$ does not involve *C*, so the *geometry* of the hazard, which setpoints are unsafe and by how much, is identical across the entire sweep. Two implementation quantities follow *C* by construction: the integrator gain $k_{i}=0.003\;C/C_{\mathrm{ref}}$, so that controller aggressiveness matches the plant, and the integration step, bounded by τ/50. Both are properties of the simulated deployment rather than free parameters, but the sweep is therefore of the plant together with its matched controller, not of the plant alone. We also vary the runtime monitor’s sampling period, because a plant slewing at 40 °C/min moves several degrees between two samples of a slow monitor and is already deep out of band by the time the runtime first looks at it; without varying both, the plant and the monitor cannot be separated.

The monitor reads a *noisy sensor*, not the true plant state, so whether a marginal excursion trips the rollback deadband is stochastic. Each point is therefore the mean of three seeds, and we report the spread.

### 9.2. Result: A Threshold, Not a Gradient

The behavior is a threshold, and that is what makes it useful:

- on plants slewing at 6.9 °C/min or faster (τ≤5.4 min), rollback removes about 95% of the unsafe exposure when the monitor samples every 1–5 s;
- on plants slewing at 1.4 °C/min or slower (τ≥27 min), it removes at most 4.8% at a 1 s monitor and never more than 14.7% at any sampling period we tested.

The dependence on the sampling period is not monotone: at τ=27 min rollback removes 4.8% at a 1 s monitor and 14.7% at 30 s. We have no established mechanism for the inversion and report it as observed; the reading carried forward is the conservative one, that below the threshold rollback never removed more than 14.7% at any period tested.

The system either wins the race against the next bad command on every cycle or it loses on every cycle, and the transition between the two is narrow. Rollback undoes *one* command, and a systematically wrong model simply issues it again on the next cycle, so the runtime must be able to restore the plant well inside the interval between decisions or it never catches up at all. The width of the seed spread is itself informative: it is ±0.0 to ±0.1 across most of the two plateaus (the exception is ±3.1 at 10.4 °C/min) and ±3 to ±4 in the transition, together with the 25–35% removed there, which is what one expects near a threshold, where the outcome of a marginal excursion is genuinely stochastic.

Two independent bounds emerge. The *upper* bound is the plant: the building of this paper (τ=3 h, 0.21 °C/min) sits far above the threshold, rollback removes 0.2%, and sampling faster does not help because the bottleneck is the room and not the sensor. The *lower* bound is the monitor: on the fastest plants a 30 s monitor is worth a fraction of a 1 s one, because the plant leaves the safe band between samples and the runtime only ever sees the aftermath. Here the bottleneck *is* the sensor, and unlike the room it is fixable.

**Figure 9:**
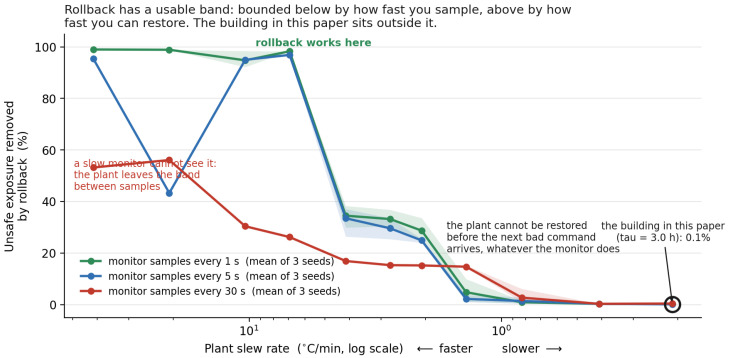
Rollback has a usable band, bounded below by how fast the runtime can sample and above by how fast the plant can be restored. Shaded regions are the seed spread. The building of this paper sits outside the band. Values are those of Table 17.

Figure 9 plots the sweep. We report one observation we cannot fully explain. The 5 s column contains a reproducible dip at 20.7 °C/min (43.3%, spread ±0.1) that is not seed noise and that we do not account for; it appears to be an interaction between the monitor’s fixed sampling period and the plant’s speed at that particular ratio. It does not affect either bound or the criterion, and we state it rather than smoothing it away.

### 9.3. The Criterion

The honest claim is therefore not that rollback does not work; it is the following:

*Runtime rollback delivers safety when the plant can be restored faster than the next bad command arrives and sampled faster than it can leave the safe set. Outside that band its contribution falls to at most 14.7% and it is better described as a bookkeeping mechanism: it still yields a correct audit trail and a defined terminal state, but not safety. Safety there must come from admission control, and admission control is only as good as the policy’s estimate of context.*

Every quantity in the criterion is measurable before any code is written: recovery time, control period, monitor period. That, rather than any number specific to our twin, is what we intend to transfer.

## 10. Robustness

Every conclusion above rests on choices we made: a model architecture, a random seed, a sensor noise level, a set of safety bands, a scoring convention. A conclusion that holds only under one of those is not a conclusion. We therefore attacked each in turn.

The result is uniform: all 18 perturbations preserve every claim they can test (53 of the 54 claim-perturbation cells; strict scoring without the grace window cannot test C3). The qualitative ordering is therefore robust to the tested architectures, seeds, noise levels, safety bands and scoring variants; we claim robustness over those tested choices, not over the space of all possible ones. In particular they survive a *linear* model, which is the strongest single piece of evidence that they are not artifacts of a particular architecture: the finding holds identically for a four-neuron network, a 128-64-32 network, a ridge regression and a random forest.

The monitor’s deadband is derived from the noise floor (five standard deviations) rather than fixed, because a constant 0.25 °C deadband is half a standard deviation at σ=0.5 °C and the monitor would fire on noise, making the experiment measure a defect in itself. With that in place the sweep is clean: exposure without rollback is *identical* (954.8 °C·min) at all three noise levels, as it must be, because the oracle scores the true plant state and measurement error does not change the truth. Sensor noise perturbs what the runtime *decides*, not what physically happened, and across a tenfold change in the noise floor it does not perturb those decisions enough to move a claim.

### 10.1. The Floor: Validating the Metric Itself

One check deserves separate statement, because it validates the instrument rather than the result. We replace the AI service with one that *cannot be wrong*, handing it the ground-truth occupancy, and ask what our metric reports.

A perfect model scores exactly 0.0 under the grace window. That is what makes the window the right instrument: it counts only what a controller could actually have avoided, and nothing else. Under strict scoring the same perfect model pays 337.0, which is the ramp cost of the 22/17 *setback strategy* rather than a governance failure, and a controller that never sets back pays none of it. The oracle gate’s 130.1 sits *below* even that, so it is not a governance failure either; it is the control strategy’s bill, and it should not be charged to the runtime. Separating those three things, governance failure, context-estimation failure, and the physical cost of the control strategy, is only possible because the oracle is independent of the gate.

### 10.2. Is the Shift Real?

We call datatest2 the distribution shift, and we should say plainly what does and does not shift. The occupancy *rate* does not: it is 21.0% in datatest2 against 21.2% in training, whereas datatest, which we call in-distribution, is at 36.5%. The shift is in the *features*: humidity and humidity ratio move by 0.8 training standard deviations and CO_2_ by 0.5. That is precisely what breaks a model which must infer occupancy *from* those features, and it is also why the CO_2_ context predicate degrades under shift while remaining as good as the oracle in distribution. The split names are the published UCI ones.

## 11. Discussion

### 11.1. Principal Findings

**Cost of the mechanism.** Policy evaluation is not the binding constraint: about 1 μs inline, a median of 0.44 ms end-to-end on the real MQTT stack, with queue contention dominating beyond roughly 1000 modeled devices (Appendix B, from a calibrated timing model rather than a live deployment).

**RQ1.** An automated, oracle-based policy gap finder ranks every single-predicate mutation by the physical exposure it admits and flags the shipped policy’s own 20.2% gap without hand-picking; the decisive result is that policy testing *without* a physical oracle detects none of these physical actuation gaps (Section 6.2). Enabling the missing G2 source predicate closes the shipped-policy gap. We do not claim to outperform static rule interaction analyzers such as iRuler; the finder answers the complementary, physical question.

**RQ2, the principal result.** Once governance decisions have physical consequences, the ordering of the mechanisms is not the one the governance literature commonly assumes. Admission control removes a substantial fraction of unsafe physical exposure (1185.3→954.8 °C·min); checkpoint rollback, added on top, removes a further 0.2% and does not reduce the peak excursion at all, because the decision costs 0.44 ms and the physical recovery a median of 61 min. These are deterministic paired outcomes on one trace and one plant, not samples from a population, so no confidence interval is attached; re-rolling the sensor noise realization over four seeds moves the additional benefit between 0.1% and 0.4% (Section 10). What removes the exposure is prevention: an oracle context predicate leaves 1.2 °C·min on the same runtime and the same plant, so the limiting factor is the policy’s estimate of context, not the runtime. The deployable CO_2_ proxy leaves 727.7 under shift and 0.5 in distribution, and that gap measures how much residual physical risk belongs to the context estimator.

A second result of RQ2 is that rejection *semantics* dominate rejection *accuracy*. An oracle-accurate gate that reverts to a context-blind held state (924.8) is beaten by a substantially less accurate gate that reverts to a context-independent safe state (727.7). A gate that only vetoes is not a controller: the actuator keeps tracking a setpoint chosen for the old context. This is invisible in a software-only testbed and is, to our knowledge, not reported elsewhere.

**RQ3.** Sweeping the plant across more than two orders of magnitude of speed converts the negative result into a criterion. Rollback removes about 95% of unsafe exposure on plants restorable faster than the command interval and at most 14.7% on plants that are not (4.8% at a fast, 1 s monitor; the dependence on the sampling period is not monotone, Table 17), with a narrow transition. Two independent bounds apply: the plant’s recovery time (which no monitor can fix) and the monitor’s sampling rate (which is fixable). Both are measurable on a real system before any code is written. Runtime rollback is therefore a safety mechanism for fast actuation and a bookkeeping mechanism for slow ones. It still yields a correct audit trail and a defined terminal state on a slow plant, and against *irreversible* actuation it yields neither safety nor recovery, only an honest FAILED_SAFE and an escalation to an operator.

We regard the negative form of these findings as their main value: the mechanisms measured here are the ones the governance literature routinely recommends, and the closest prior family, Simplex-style runtime assurance and restart-based recovery [18,20], asks which controller to engage next, whereas the per-mechanism accounting of physical risk asked here is complementary and does not appear to have been reported in this form.

### 11.2. Applicability Boundary

The measured claims of this paper are scoped to the studied smart building climate loop. Environmental monitoring and industrial platforms in which sensors trigger equipment control are candidate settings for the same mechanisms, but only the criterion of Section 9, and not the exposure numbers, should be transferred to them. In these settings a microsecond-scale gate evaluation and a sub-millisecond decision path are acceptable when they buy auditability and admission control. The criterion of Section 9 makes the boundary for the *rollback* component precise, and it is narrower than the boundary for the runtime as a whole: on a slow thermal plant, rollback should be budgeted as an accountability mechanism and not as a safety one, and the safety budget should be spent on the policy’s context instead. The design is not positioned for hard real-time actuation loops such as low-level vehicle control or protective relay tripping; there, the governance runtime belongs at the supervisory layer rather than inside the certified control loop.

### 11.3. Relation to Policy-as-Code Practices

The backend comparison complements existing policy-as-code practice. OPA and Kubernetes Gatekeeper demonstrate that policy evaluation can be embedded in deployment admission [12,13]. Relative to IoT runtime platforms such as EdgeX Foundry and KubeEdge, the proposed runtime is not a replacement but a governance layer that would sit above their device-management and messaging services and gate sensor-originated intents before actuation; the intent lifecycle coupling of admission, rollback, and hash-linked audit is precisely what those platforms leave to application code. The present work extends the evaluation into the IoT control lifecycle and, more importantly, measures what the enforcement is worth once the enforced system is physical. The EU AI Act emphasizes runtime oversight and lifecycle governance [11]; executable gates and hash-linked audit records provide technical mechanisms that can support such evidence. Our results add a caution to that program: an audit trail proves what happened, and on a slow plant that is most of what a rollback mechanism can honestly offer.

### 11.4. Threats to Validity

*Internal validity.* No physical hardware was operated. Every physical quantity in Section 8, Section 9 and Section 10 is produced by the digital twin of Section 8.1, whose model and parameters are given in full so that they can be challenged. The twin is a lumped single-zone RC model; it does not represent stratification, multi-zone coupling, solar gain, or moisture transport. The sweep of Section 9 is the direct response to the objection that the conclusions might be artifacts of these parameters: it varies plant speed across more than two orders of magnitude and reports where the conclusions change and where they do not.

*Metric validity.* The grace window could in principle be an instrument that flatters the governed arms. It is not: under a perfect model, grace-scored exposure is exactly 0.0 (Table 18), which is precisely the property it is supposed to have, and the ungoverned arm still scores 1185.3 through it. Scoring without the window preserves C1 and C2 (Table 19); C3 is not meaningful under strict scoring because it would be asking the gate to repeal thermodynamics, and we do not make that claim.

*Construct validity.* The safety oracle is ours, and its bands are a judgement call. We therefore vary them: four reasonable choices, including tighter and looser and asymmetric bands, preserve all three claims. The independence of the oracle from the gate is what makes the false negative measurements non-circular, and it is enforced structurally: the oracle reads the true plant state and the true occupancy, and neither is available to the governance layer.

*External validity.* The AI failure and closed-loop results use a single publicly available occupancy dataset. Six model architectures, including a linear one, produce the same conclusions, so the findings are not artifacts of the learner; they may nevertheless be artifacts of this building. Replication on a second building is the most valuable single extension of this work, and we identify it as such rather than claiming coverage we do not have. The evaluation also uses a single governance-policy structure with fixed thresholds; more complex predicate logic could produce different overhead profiles.

*An unexplained observation.* The 5 s monitor curve in Table 17 contains a reproducible dip at 20.7 °C/min that is not seed noise and that we cannot fully account for. It affects neither bound nor the criterion. We report it because it is there.

*Scope limitations.* The implementation does not include formal verification of the state machine properties. The safety and liveness invariants of Appendix A are stated as design invariants supported by the transition function structure, not as formally proved theorems. The physical actuation latency is not measured: the released GPIO backend implements it, and a mechanical parity check confirms that the governance code path is identical in simulation and on the Raspberry Pi, but the backend was not executed, and no number in this paper depends on pretending otherwise.

*Recovery-path idealization.* The checkpoint eligibility and monitor predicates are instantiated from the evaluation testbed’s context-dependent bands, whose occupancy argument is the ground-truth label (Section 3.6). The admission gates never see it, so the false negative measurements remain non-circular, but the rollback arm is measured under oracle-assisted context selection rather than under a deployable estimator, and the magnitude and direction of the resulting bias were not quantified. What the experiment establishes is narrower and still decisive: even with ground-truth context available to the recovery predicates, rollback removed only 0.2% of unsafe exposure on the evaluated slow plant.

### 11.5. Future Work

*Physical deployment* is the outstanding item. The hardware abstraction layer is complete on both sides and parity-checked, so the hardware evaluation is an interface swap rather than a rewrite: Raspberry Pi 4 with an SCD40 CO_2_ sensor, a BME280, an opto-isolated relay and a servo, which would supply the physical actuation latency, the power consumption figures, and a live demonstration of the CO_2_ predicate on a real sensor. The criterion of Section 9 predicts which rollback benefit regime that such a deployment falls in and can be refuted by it; effects it does not model, including live sensor noise, actuator dynamics, power, and operating system jitter, are exactly what that rung would add.

*A second building.* The single-dataset dependence is the clearest external validity limit, and repeating the study on an independent occupancy dataset is the most valuable next experiment.

*Closing the residual.* Our results locate the residual risk in the context estimator rather than in the runtime, which redirects effort: gates that consume richer context (zone, provenance, occupancy estimates with confidence), and that are themselves evaluated under distribution shift, since we show the context signal shifts too. *Other directions* include richer policy structures, a broader adversarial taxonomy, lightweight model checking of the state machine invariants, and adapter layers for EdgeX and KubeEdge.

## 12. Conclusions

We asked which runtime governance mechanisms actually reduce physical risk in an AI-driven IoT system, and under what conditions, and we built a closed loop in which the question has an answer. A real setpoint model, trained on real occupancy data, drives a physics-based thermal plant through a declarative governance plane, and every arm is scored by an independent, context-dependent safety oracle that the admission gates can neither observe nor influence.

The answer inverts the commonly assumed ordering. Admission control removes a substantial fraction of the unsafe physical exposure; checkpoint rollback, added on top, removes a further 0.2% and does not lower the peak excursion, because the room recovers orders of magnitude slower than the governance decision that governs it. What removes exposure is prevention, not recovery, and the residual that prevention cannot reach belongs to the policy’s estimate of context rather than to the runtime. Rejection semantics matter more than rejection accuracy: what a gate does when it says no dominates how often it is right.

Sweeping the plant across more than two orders of magnitude of speed turns this into a transferable criterion: runtime rollback is a safety mechanism for fast actuation and a bookkeeping mechanism for slow ones, and which regime a system is in can be read from its recovery time and sampling rate before any code is written.

These conclusions survive across model architectures (including a linear one), seeds, noise levels and safe-band choices, and a model that cannot be wrong scores exactly zero under our metric, which shows the metric counts only what a controller could have avoided. We stopped at the third rung of the evaluation ladder: **no physical sensor or actuator hardware was operated**, so this is software-in-the-loop rather than hardware-in-the-loop. The parity-checked Raspberry Pi backend, the trained model, all experiments and the tests are released, and the results were reproduced on three computational environments spanning Windows 10, Windows 11 and Ubuntu 22.04, so the fourth rung is an interface swap, and our criterion makes a testable prediction about its outcome.

## Figures and Tables

**Table 1:** Functional, scope-based positioning. This is not a performance benchmark: these systems do not implement the same governance-gate, rollback, and audit semantics, and the static analysis tools reason over rule interactions our finder does not model, while our finder measures the physical exposure a policy omission admits. The comparison locates this work, it does not claim superiority.

System	Policy Admission	IoT Lifecycle	Rollback	Audit Chain	Policy Gap Detection
OPA/Gatekeeper [12,13]	Yes	Deployment adm.	No	No	No
EdgeX/KubeEdge [21]	Partial	Yes	Limited	Logs	No
Soteria/iRuler/HomeGuard [3,7,8]	N/A	Analysis only	No	No	Yes (deeper)
IoTGuard/IoTSafe [5,6]	Yes (runtime)	Yes	No	Logs	Partial
This testbed	Yes	Intent lifecycle	Software checkpoint	Hash-linked	Automated (oracle-based)

**Table 2:** Governance runtime states and allowed transitions.

State	Description	Allowed Next States
IDLE	No active governance operation	ADMITTING, THROTTLING
ADMITTING	Intents checked against G1–G4	EXECUTING, REJECTING, THROTTLING
EXECUTING	Dispatched to AI services and devices	COMMITTING, ROLLBACK
COMMITTING	State, checkpoint, and audit finalized	IDLE
THROTTLING	Non-critical intents delayed	ADMITTING, ROLLBACK
ROLLBACK	Latest eligible checkpoint restored	IDLE, FAILED_SAFE
FAILED_SAFE	Operator intervention required	-

**Table 3:** Policy gate specifications: evaluated attributes and acceptance criteria.

Gate	Evaluated Attributes	Acceptance Criteria	Rejection Action
G1 Safety	Setpoint value, action type, device capability	Setpoint within $\left[T_{\min},T_{\max}\right]$; action ∈ allowed set; device supports action	Reject intent
G2 Privacy	Timestamp, source ID, device descriptor	Timestamp field non-null and well-formed. Note: the current implementation checks timestamp presence but does not validate the source field, which is the root cause of the partial corruption bypass (20.2% for the shipped policy, Section 6.2; 18.7% for the timestamp-only variant, Appendix B).	Reject or flag for review
G3 Resilience	Queue depth, service health, replica status	Queue depth $<Q_{\max}$; service responsive within timeout; ≥1 healthy replica	Throttle or defer
G4 Auditability	Intent ID, sequence number, device ID	Monotonic sequence; valid device registration; no duplicate intent ID	Reject and log anomaly

**Table 4:** Failure model and governance response.

Failure Type	Description	Response
Service crash	AI inference or orchestration unavailable	Checkpoint restoration
Queue overload	Bounded threshold exceeded	Intent throttling
Policy violation	Safety, privacy, or resilience constraint violated	Rejection or rollback
Replica inconsistency	Temporary divergence under partition	Synchronization before commit
Runtime anomaly	Drift or abnormal behavior detected	Mitigation or rollback

**Table 5:** Software environment that reproduced the reported results; the full pinned list (requirements-lock.txt) accompanies the code.

Component	Version/Setting
Python	3.10 (3.10.20 under Windows 10; 3.10.12 under Linux)
Numerical/ML stack	numpy 2.2.6, scipy 1.15.3, pandas 2.3.3, scikit-learn 1.7.2
Plotting/config	matplotlib 3.10.9, pyyaml 6.0.2
Messaging	paho-mqtt 2.1.0; embedded broker amqtt 0.11.3 for the self-contained demo
Real-stack services	Eclipse Mosquitto 2 broker; in-process policy decision point (measured). An OPA Rego decision point is also provided via docker compose
Testing	pytest 9.1.1 (25 tests)
Dataset	UCI Occupancy Detection [26]; light channel excluded; the four features standardized on the training split only
Failed/incomplete runs	Discarded and re-run, never partially scored; no missing value1*uditchain*1udit chain imputation

**Table 6:** The evaluation ladder, in the standard cyber-physical sense. We occupy the first three rungs and state plainly that we do not occupy the fourth. Italics mark what is identical to the rung above; bold marks the components that would be physical rather than simulated.

Rung	Controller	Plant/Environment	This Paper
Simulation (Tier 1)	governance code	abstract workload, modeled timing	Section 6
SIL (Tier 2)	*the same* governance code	physics-based plant model, real sensor traces, real learned model	Section 8, Section 9 and Section 10
Prototype (Tier 3)	*the same* governance code on a real MQTT bus with an in-process policy decision point	real broker, real network stack, real disk	Section 7
HIL (Tier 4)	the same governance code on a Raspberry Pi	**real sensors and actuators**	**not performed**

**Table 7:** Policy backend comparison (30 seeds, 200 devices, 10% fault rate).

Backend	Policy P90 (ms)	E2E P90 (ms)	MTTR P90 (ms)	Admit Rate
Inline	0.001	5.90 [5.82, 5.96]	1.28	0.920
HTTP REST	0.99 [0.96, 1.01]	6.86 [6.74, 6.98]	1.28	0.920
Subprocess CLI	25.95 [25.18, 26.71]	31.31 [30.48, 32.12]	1.28	0.920

**Table 8:** Fault detection recall on a six-fault battery (30 seeds, bootstrap 95% CIs). Only methods with an independent physical safety oracle detect physical actuation policy gaps; rule-level testing without one detects nothing at any budget. Random search with the oracle matches the coverage-guided finder on this battery: the oracle, not the search strategy, is the decisive ingredient. Bold marks the method proposed in this paper.

Method	Recall	95% CI
Manual injection (one hand-picked predicate)	0.17	[0.17, 0.17]
Policy-consistency testing (no physical oracle)	0.00	[0.00, 0.00]
Random intents + physical oracle	1.00	[1.00, 1.00]
**Coverage-guided + physical oracle (ours)**	**1.00**	**[1.00, 1.00]**

**Table 9:** Specification of the learned setpoint model and its generalization. All values are produced deterministically by the released full_training.py and were reproduced independently on a second machine and operating system. The rising error across splits is the distribution shift the paper studies, not a tuning artifact.

Property	Value
Architecture	MLP, hidden layers (32, 16), ReLU, Adam, fixed seed
Learned parameters	705
Training data	UCI datatraining.txt, 8143 rows (1729 occupied/6414 vacant)
Features	Temperature, Humidity, CO_2_, HumidityRatio (standardized; light excluded)
Target	22 °C occupied, 17 °C vacant setback
Convergence	327 iterations; loss 178.7→0.34
Training error (MAE)	0.40 °C
Occupancy recovery	96.4% at the 19.5 °C midpoint
MAE, train/held-out/shifted	0.40/1.69/3.32 °C

**Table 10:** Real AI service on real sensor data [26]. Residual risk is the share of physically unsafe commands that the gate admits. Nothing is hand-injected: all errors are the learned model’s own.

Regime	Setpoint Range (°C)	Out of Band	Blind-Gate Residual	+CO_2_ Residual	Gate AUC
In distribution	[16.7,24.5]	0.0%	100.0%	15.7%	0.553
Distribution shift	[13.8,40.9]	7.1%	48.0%	44.2%	0.863

**Table 11:** Scalability analysis (30 seeds).

Devices	E2E P90 (ms)	Policy P90 (ms)	MTTR P90 (ms)
10	5.74 [5.62, 5.86]	0.001	1.14 [1.09, 1.22]
100	5.83 [5.76, 5.90]	0.001	1.29 [1.26, 1.30]
500	6.35 [6.27, 6.43]	0.001	1.30 [1.29, 1.31]
1000	79.79 [79.53, 80.05]	0.001	1.30 [1.28, 1.30]
2000	105.78 [105.64, 105.92]	0.001	1.30 [1.28, 1.31]
5000	105.78 [105.64, 105.92]	0.001	1.29 [1.28, 1.30]

**Table 12:** Governance plane measured as a deployed service on a real MQTT stack with an in-process executable policy decision point, the same gates additionally evaluated through a real Open Policy Agent server over HTTP, and a disk-backed hash-chained audit log. No timing in this table is modeled. In-process latencies are reported for two hosts, and the OPA HTTP path for the Windows host.

Quantity (Real MQTT Stack)	Value
Control intents published	240
Admitted/rejected by the gates	120/120
Commands applied by the actuator	120
Governance decision latency, Windows host	median 0.44 ms, P90 0.64 ms
Governance decision latency, Linux host	median 0.09 ms, P90 0.34 ms
Governance decision via OPA HTTP, Windows host	median 0.94 ms, P90 1.05 ms
Persistent audit records/chain intact	240/yes
Chain verifies after one record altered on disk	no (tamper detected)
Forged provenance admitted, shipped policy	40/40
Forged provenance admitted, corrected policy	0/40

**Table 13:** Unsafe physical exposure under distribution shift (datatest2, 9752 min). Same model, same trace, same plant; only the governance changes. Values are deterministic paired outcomes, not samples from a population; the 0.2% rollback benefit ranges from 0.1% to 0.4% across four sensor noise seeds (Section 10). Scored by the independent oracle against the true plant state.

Governance	Unsafe Exposure (°C·min)	Peak Excursion (°C)	Rollbacks
Ungoverned	1185.3	3.75	0
Admission control only	954.8	3.75	0
+checkpoint rollback	953.2	3.81	9
+CO_2_ context predicate	727.7	3.81	5
Oracle context (not deployable)	1.2	0.16	1

**Table 14:** The two halves of rollback. The decision is six orders of magnitude faster than its own physical consequence.

Quantity	Value
Governance decision (measured on the real MQTT stack)	0.44 ms
Physical recovery, median	3640 s (61 min)
Physical recovery, worst case	8760 s (146 min)

**Table 15:** Learned against deterministic control under identical governance. This is a *system-level* comparison, not an isolated comparison of controller robustness: the gate intervenes far more often on the learned controller’s output, so each row reflects controller behavior and governance interaction. The rule matches the MLP in distribution but degrades under shift, because its single context signal shifts; the no-setback controller incurs zero unsafe exposure under the temperature oracle and carries the highest heating demand. Exposure in °C·min; heating demand proxy in kWh-equivalent over each trace. Bold marks the value discussed in the accompanying text.

Controller	Unsafe Exposure		Heating Demand	
	**In-Dist.**	**Shift**	**In-Dist.**	**Shift**
MLP (learned, 4 features)	221.5	**953.2**	27.7	115.6
CO_2_-rule thermostat	200.4	1427.7	23.4	83.6
Constant 22 °C (no setback)	0.0	0.0	38.8	144.7

**Table 16:** Three rejection semantics, applied to the same policies on the same trace. hold keeps the current setpoint; checkpoint reverts to the last setpoint that provably held the room in band; safe_state commands 21 °C, which lies inside *both* bands and is therefore safe without knowing which one applies. Bold marks the lowest unsafe exposure in the table, which is the configuration the surrounding discussion is about.

Policy	Rejection Semantics	Unsafe Exposure (°C·min)
Oracle	hold	924.8
Oracle	checkpoint	918.7
Oracle	safe_state	**1.2**
Corrected	hold	734.0
Corrected	checkpoint	727.9
Corrected	safe_state	727.7

**Table 17:** Fraction of unsafe exposure removed by rollback, as a function of plant speed and monitor sampling period. Mean of three seeds; ± is half the observed spread. The building of Section 8.1 is the last row.

τ (h)	Slew (°C/min)	Monitor 1 s	Monitor 5 s	Monitor 30 s
0.015	41.4	99.0±0.0	95.4±0.1	53.2±0.0
0.030	20.7	98.9±0.0	43.3±0.1	56.1±0.1
0.060	10.4	94.8±3.1	95.0±0.1	30.4±0.1
0.090	6.9	98.3±0.0	96.9±0.1	26.2±0.1
0.150	4.1	34.5±4.2	33.5±5.3	16.9±0.2
0.225	2.8	33.2±3.2	29.6±4.0	15.3±0.1
0.300	2.1	28.7±4.2	24.9±0.8	15.2±0.2
0.450	1.4	4.8±4.3	2.2±1.4	14.7±0.3
0.750	0.8	0.9±0.5	1.4±1.2	2.7±2.6
1.500	0.4	0.3±0.1	0.3±0.1	0.3±0.0
3.000	0.2	0.2±0.1	0.1±0.1	0.4±0.2

**Table 18:** The floor. A model that cannot be wrong must score zero under a metric that counts only what a controller could have avoided. Bold marks the zero-exposure entries, on which the floor argument depends.

Controller	Grace-Scored	Strict
	**(°C·min)**	**(°C·min)**
Perfect model, 22/17 setback	**0.0**	337.0
Constant 22 °C, no setback	**0.0**	0.0
Oracle gate, real (failing) model	1.2	130.1

**Table 19:** Robustness of the three central claims. C1: rollback removes less than 10% of unsafe exposure. C2: the corrected predicate beats the shipped policy. C3: the oracle predicate removes essentially all of the exposure a controller could have avoided.

Perturbation	C1	C2	C3
6 model architectures (4 neurons to 128-64-32; ridge; random forest)	holds	holds	holds
4 random seeds	holds	holds	holds
3 sensor noise levels (σ=0.05,0.25,0.5 °C)	holds	holds	holds
4 sets of safety bands	holds	holds	holds
Scoring without the grace window	holds	holds	n/a ^a^

^a^ Not a meaningful test on strict exposure; see the floor argument below.

## Data Availability

The complete reproducibility package (runtime scripts, the three policy backends, the OPA Rego encoding, configuration seeds, all generated traces, and the statistical analysis and verification scripts) is provided as Supplementary Materials and is publicly available at https://github.com/saydirasulov-alt/governance-runtime-iot (accessed on 27 July 2026), permanently archived on Zenodo (concept DOI: https://doi.org/10.5281/zenodo.21374935 (accessed on 27 July 2026); this release, v1.2.0, Git commit 34a5fcd, which is the state that produced every reported number: https://doi.org/10.5281/zenodo.21557066 (accessed on 27 July 2026)). Every reported table is regenerated by documented commands shipped with the release: run_sil.py (closed loop), run_all.py (sweep and robustness), run_governance_demo.py (Tier-3 real stack) and reproduce_all.py (Tier-1 tables and audit chain); the release README gives the exact invocation, the pinned dependency list and the expected outputs. Deterministic quantities reproduce bit-for-bit under the pinned environment; live latencies are host-dependent and are remeasured by the same scripts.

## Supplementary Materials

The following supporting information can be downloaded at https://www.mdpi.com/article/10.3390/s26154921/s1. The archive contains the reproduction record of the paper and the software that produces it. **Reproduction record** (folder reproduction/): File S1: MASTER_REPRODUCTION_LOG.txt, the complete machine-generated transcript of an end-to-end reproduction, ending in the result line 127 PASS/0 FAIL; File S2: REPRODUCTION_EVIDENCE.md, the software environment, the per-experiment breakdown of the 127 checks and the host-dependent latencies; File S3: CLAIM_AUDIT.md, a claim-by-claim audit of the manuscript against the released code; File S4: RUN_ALL_LOG.txt, the closed-loop, plant-sweep and robustness log; File S5: REPRODUCTION_LOG.txt, the Tier-1 tables, ablations, audit-chain verification and Open Policy Agent conformance log; File S6: OPA_HTTP_LOG.txt with opa_http_latency.csv, the live Open Policy Agent decision latencies over 240 requests; File S7: OPA_SERVER_LOG.txt, the Open Policy Agent v1.18.2 server log; File S8: verify_paper_numbers.py, the script that checks every reported value; File S9: README.txt, an index of the record. **Software** (archive root): File S10: governance_runtime.py, the runtime with gates G1–G4, checkpointing and the hash-chained audit log; File S11: the three policy backends, policy_loader.py, policy_http_server.py and policy_cli_evaluator.py, with opa_conformance.py; File S12: the policy definitions, policy_config.json, policy_config.yaml, policy_config_corrected.json, policy_config_corrected.yaml and policy_gates.rego; File S13: the experiment driver and statistical analysis, experiment_runner.py, experiment_config.json and stats_analysis.py; File S14: the auxiliary experiments, ai_failure_characterization.py, gate_complexity_benchmark.py, rollback_demo.py and hardware_rollback_harness.py; File S15: the verifiers and unit tests, verify_audit_chain.py, verify_corrected_g2.py and test_policy_loader.py; File S16: the documentation, README.md, HARDWARE_README.md, ISH1_config_loader_notes.md, ISH2_ai_failure_notes.md, ISH3_rollback_notes.md and table12_ai_failure.md; File S17: requirements.txt, the pinned dependencies; File S18: folder results/, the generated traces 01_backend_comparison.csv, 02_ablation.csv, 03_baseline.csv, 04_network_sensitivity.csv, 05_adversarial.csv, 06_scalability.csv, ai_failure_roc.csv and rollback_mttr_ms.csv, with table12_ai_failure.md and the two generated figures fig_ai_failure_roc.png and fig_rollback_mttr.png.

## Appendix A. Formal Governance State Model

The runtime is formalized as a state transition system G=(S,I,Π,δ,R,C,A) over governance states *S*, intents *I*, the executable policy set Π, transition function δ, rollback operator *R*, checkpoint set *C* and audit log *A*. An intent is admitted only if every mandatory predicate holds, $\mathrm{Pass}\left(i,\Pi \right)={⋀}_{\pi_{j}\in \Pi }\pi_{j}\left(i\right)=1$. Checkpoints carry two predicates: $\mathrm{Valid}(c_{j})$ holds when the audit entry has been appended and the hash chain is consistent, and

$$\mathrm{Eligible}\left(c_{j}\right)=\mathrm{Valid}\left(c_{j}\right)\wedge \mathrm{Acknowledged}\left(c_{j}\right)\wedge \mathrm{Stabilized}\left(c_{j}\right),$$

where Acknowledged records actuator acceptance and Stabilized is the predicate of Section 3.6. Rollback restores the latest *eligible* checkpoint, $R\left(s_{t}\right)=c_{k}$, $k=\max\{j\leq t:\mathrm{Eligible}\left(c_{j}\right)=1\}$; provisional records satisfying Valid alone are never selected.

Two design invariants are exercised by the test suite rather than proved. **Safety**: an intent violating a mandatory predicate cannot reach COMMITTING, because δ permits ADMITTING → EXECUTING only when all mandatory predicates hold. **Liveness**: every detected violation terminates in IDLE after rollback or in FAILED_SAFE after escalation, given at least one eligible checkpoint and an available rollback controller. Measured recovery obeys $T_{\mathrm{MTTR}}\leq T_{d}+T_{g}+T_{r}+T_{c}+\varepsilon_{q}$, each term taken from runtime timestamps. The full transition table, checkpoint record structure, per-intent commit atomicity argument, and the digital twin rollback controller statistics are given in the artifact.

## Appendix B. Tier-1 Supporting Characterizations

The Tier-1 synthetic characterizations (policy backend overhead across inline, HTTP-style and subprocess-style evaluation, gate complexity sensitivity, the device count scalability curve and the M/M/1 queue pressure model that generates it) support the gateway-side cost of the mechanism and underpin no physical-risk claim. To keep this paper focused on the closed-loop measurement, their tables, figures and parameters are provided in full in the supplementary artifact, which regenerates each of them. The two facts the body relies on are that inline policy evaluation costs ≈1 μs while an HTTP decision point adds ≈1 ms and a subprocess decision point ≈26 ms at identical admission decisions, and that end-to-end latency is bounded by queue contention beyond roughly 1000 modeled devices rather than by policy evaluation; both are outputs of a calibrated timing model on a gateway class node, not live deployment measurements.

## Appendix C. Corrected-G2 Ablation

**Table A1:** Corrected-G2 ablation: adding a source-field predicate closes the partial corruption bypass at the policy-specification level (30 seeds, 20% fault rate).

G2 Configuration	Partial Corruption Unsafe Admission	Detection Rate
Timestamp only (evaluated)	18.7%	81.3%
Timestamp + source (corrected)	0.0%	100%
